# Enhancing tomato quality, high sugar content and GABA accumulation, with mutations in *ESKs* and *GAD3* genes

**DOI:** 10.1038/s41598-025-28888-5

**Published:** 2025-12-02

**Authors:** Seungje Choi, Takeru Iwama, Misaki Kobayashi, Islam M. Y. Abdellatif, Hiroshi Ezura, Kenji Miura

**Affiliations:** 1 Graduate School of Life and Earth Sciences, University of Tsukuba, Tsukuba, 305-8572 Japan; 2 Tsukuba-Plant Innovation Research Center, University of Tsukuba, Tsukuba, 305-8572 Japan; 3 Department of Horticulture, Faculty of Agriculture, Minia University, El-Minia, 61517 Egypt

**Keywords:** *SlESK1-3*, *SlGAD3*, High-sugar, GABA-rich, High-vitamin C, Drought tolerance, Biotechnology, Genetics, Molecular biology, Plant sciences

## Abstract

Tomatoes are among the most widely cultivated crops worldwide, and their nutritional value has recently gained attention in the context of functional foods. In this study, we developed tomato varieties with elevated sugar content and high gamma-aminobutyric acid (GABA) levels using a genome editing technique to introduce mutations in *SlESK* and *SlGAD3*, aiming to enhance the nutritional value of tomatoes. Tomato plants possess three *ESK* homologs. We successfully generated tomatoes with mutations in all three *SlESK* genes and a separate line with a mutation in *SlGAD3*. Compared to the wild type (WT), which exhibited a sugar content of approximately 4.3% Brix, the mutants showed a significantly higher sugar content of approximately 7.9% Brix. GABA accumulation in the mutants was approximately five times higher than in WT. Additionally, the mutants contained 1.5 times more vitamin C than WT and demonstrated enhanced drought stress tolerance. Delayed plant growth and reduced yield were also observed in the genome-edited plants. Transcriptome analysis revealed that the expression of genes which are involved in vitamin C biosynthesis, the auxin signaling pathway, and ethylene biosynthesis were altered in the genome-edited plants. These results may contribute to cultivating high-quality tomatoes, which are expected to be in high demand.

**Supplementary Information:**

The online version contains supplementary material available at 10.1038/s41598-025-28888-5.

## Introduction

Tomatoes are among the most widely cultivated crops globally, with an annual production of approximately 189 million tons^1^. Additionally, research has shown that tomatoes contain numerous functional compounds, making them a vital crop for supporting and promoting health^2^. Tomatoes are also consumed as fruits, and innovative breeding techniques are being employed to develop tomato varieties with high sugar content^3^. It is expected that future advancements will lead to tomato varieties with both enhanced sugar content and enriched functional compounds. For instance, a high-GABA tomato, developed through genome editing, is already commercially available^4^. Gamma-aminobutyric acid (GABA) is an inhibitory neurotransmitter in the mammalian central nervous system. GABA is also widely used as a dietary supplement due to its potential to lower blood pressure and reduce anxiety^5^.

The genes targeted for the development of tomatoes with high GABA and sugar content are *SlGAD3* (*glutamate decarboxylase 3*) and *SlESK1-3* (*Eskimo1-3; Solyc03g096030*, *Solyc05g052540*, *Solyc06g051350*).

*SlGAD3* encodes glutamate decarboxylase 3, a key enzyme in GABA accumulation in tomato fruits. *GAD3* facilitates GABA synthesis by catalyzing the removal of carboxyl group from glutamic acid. This enzyme also contains an autoinhibitory domain near its C-terminus, which stabilizes the enzyme in an inactive form under normal conditions. However, during stress responses, rising intracellular calcium ion levels trigger the formation of a calcium-calmodulin complex, which binds to the calmodulin-binding site within the autoinhibitory domain, activating *GAD3*. Previous studies have shown that genome editing of the *SlGAD3* autoinhibitory domain can successfully increase GABA accumulation in tomato fruits^6,7^.

*SlESK1-3* are homologs of the *AtESK1* gene in tomato, which has been identified as a freeze tolerance-related gene in *Arabidopsis thaliana*. The *Atesk1* mutants in Arabidopsis exhibit increased soluble sugar content and enhanced freeze tolerance under cold stress conditions^8,9^. Additionally, *AtESK1* has been shown to function as a xylan acetyltransferase in Arabidopsis. Mutations in *AtESK1* result in decreased secondary cell wall thickness and reduced stem strength^10^. In contrast, few studies have reported on *esk* mutants in other crops, particularly fruiting crops such as tomato. In this study, we aimed to generate *slesk* mutants in tomato and characterize the function of *SlESK*.

Genome editing technology is anticipated to play a significant role in future breeding efforts. This technology involves inducing DNA double-strand breaks (DSBs) at specific genomic locations using artificial nucleases that recognize and cleave target sequences. Mutations arise from errors in the DNA repair process following cleavage, enabling precise modification of endogenous genes. Genome editing can be applied to a wide range of species. Prominent genome editing tools include zinc finger nucleases, transcription activator-like effector nucleases, and clustered regularly interspaced short palindromic repeats (CRISPR)/Cas9^11^.

In this study, we utilized CRISPR/Cas9, currently the most widely adopted genome editing tool. CRISPR/Cas9 is derived from the immune defense system widely conserved in eubacteria and archaea. It consists of the endonuclease Cas9 and a guide RNA (gRNA). During the induction of DSBs, the gRNA, containing a sequence complementary to the target site, recognizes and binds to the target DNA sequence. The Cas9 protein, which possesses two DNA cleavage domains, then cleaves the DNA at the target site, generating a DSB. Target sequence recognition by Cas9 requires the presence of a protospacer adjacent motif (PAM) sequence, such as NGG in the case of SpCas9. CRISPR/Cas9 is widely used due to its advantages, including simplicity in design and construction, as well as the ability to edit multiple genes simultaneously. Indeed, genome edited foods, such as high-GABA tomatoes, have already been commercially launched in Japan^4^.

In this study, we aimed to simultaneously modify *SlESK1-3* and *SlGAD3* to enhance the value of the original tomato cultivar. Additionally, we investigated the phenotypes of the *slesk1-3* mutants to characterize the function of *SlESK*. In addition to our genome editing approach, previous studies have highlighted the importance of invertase genes in sugar metabolism under stress^12^ and identified gene families involved in ascorbic acid accumulation^13^, which collectively underline the complex regulation of these nutritional traits.

## Results

### Generation of *esksgad3* mutants using crispr/cas9

To generate tomato plants harboring T-DNA with CRISPR/Cas9, 159 regenerated plants were obtained through tissue culture of cotyledon fragments. Of these, 38 individuals were selected for sequencing after preliminary screening using MultiNA, and mutations at the target sites were confirmed in 10 individuals (Supplementary Table 1). Additionally, multiple mutations were detected in some individuals, indicating the occurrence of chimeric mutations.

**Fig. 1.**
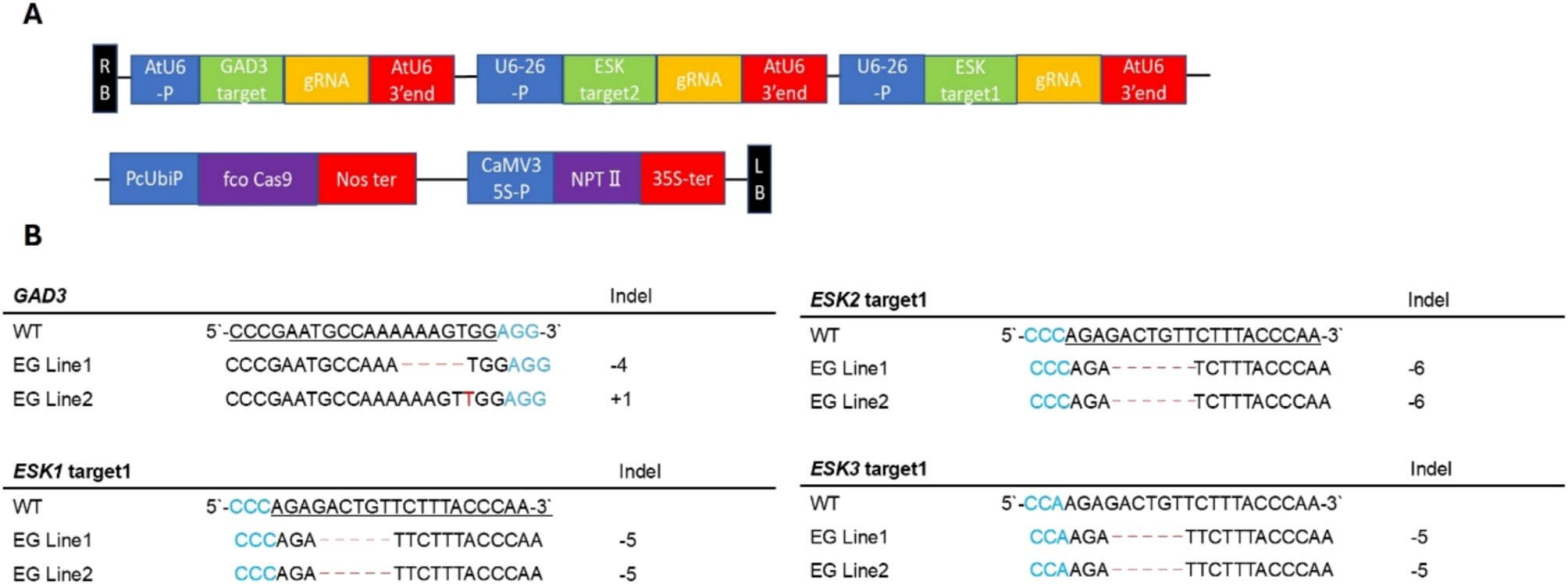
Vector structure and mutation status of eskgad3 mutant (EG Lines). (**A**) Vector map for targeting *SlESK 1–3* and *SlGAD3*. RB: Right boarder, LB: Left boarder. Promoters are indicated by blue box and terminator are indicated by red box. Each target gene is marked green box and gRNA is colored by yellow box. NPT*II* represents kanamycin resistance gene. (**B**) Each mutant sequence. (-) indicates deletions and red letter indicates the insertion. The number of deletions or insertion. Blued fonts indicate PAM sequences.

In the T_1_ generation, the inheritance of mutations at the target sites was confirmed in five lines of *esksgad3* (Supplementary Table 2). Among these individuals, homozygous mutations were successfully introduced in all target genes (*ESK1-3* and *GAD3*) in *esksgad3* line #127–5 (EG Line 1) (Fig. 1, Supplementary Table 2). Furthermore, in *esksgad3* line #127–9 (EG Line 2), although mutations were observed in the *ESK2* target and other target sites, several fully homozygous individuals were obtained in the subsequent T_2_ generation (Supplementary Table 3). Since *esksgad3* #127–9-4 exhibited severe growth retardation, and no subsequent generation was obtained, it is suggested that severe mutations in three *SlESK* homologs may cause significant developmental damage in tomatoes. Therefore, the six-base deletion in *ESK2* was selected for further characterization (Fig. 1B).

We also compared the amino acid sequence of *GAD3* and *ESK1* among the Arabidopsis’s orthologs (*AtGAD3*, At2g02000; *AtESK1*, At3g55990) and Wild-type tomato’s (SlGAD3, *Solyc01g005000*; *SlESK1*, *Solyc03g096030*; *SlESK2*, *Solyc06g051350*; *SlESK3*, *Solyc05g052540*) and each EG muants (EG Line1, EG Line2, *esksgad3* #127–9-4). GAD3 has a Ca^2+^/calmodulin-binding domain (CaMBD) at the C-terminus, and several conserved motifs are present, especially tryptophan residue and arginine (Supplementary Fig. 1)^14^. *AtGAD3* and *SlGAD3* in the WT have these motifs, but EG Line1, EG Line2, *esksgad3* #127–9-4 don’t.

AtESK1 has three annotated domains: Transmembrane helix (TM) domain, Trichome birefringence-like (TBL) domain and DUF231 domain^15^. And TBL domain has GDS motif and DUF231 domain has DXXH motif. The Ser (Serine) residue in the GDS motif together with the His (Histidine) and Asp (Asparagine) residues in the DXXH motif form a Ser-His-Asp catalytic triad required for acetylation^15^. *SlESK* 1–3 of WT conserved these domains and motifs, while in the EG mutant, SlESK2 is conserved, but SlESK1 and SlESK3 are not. And *esksgad3* #127–9-4 is not conserved three *SlESKs* (Supplementary Fig. 1).

Off-target mutations were investigated in the *esksgad3* #127 line, a genome-edited line, by comparing sequencing results with those of the wild type (WT). No mutation was detected at the predicted off-target candidate sites in any of the analyzed individuals (Supplementary Table 4). Additionally, amplification of the vector plasmid using 11 sets of primers revealed no residual foreign genes in *esksgad3* #127–5–6 (a sibling of EG Line 1) or *esksgad3* #127–9–10, #127–9–14, #127–9–18, #127–9–26, and #127–9–29 (siblings of EG Line 2) (Supplementary Fig. 2).

### Growth and fruit set impairment in eg mutants

In EG Line 2, ESK2target1 exhibited a heterozygous mutation in the T_1_ generation. Therefore, sequence analysis of ESK2target1 was performed in the T_2_ generation, with results presented in Supplementary Table 3.

**Fig. 2:**
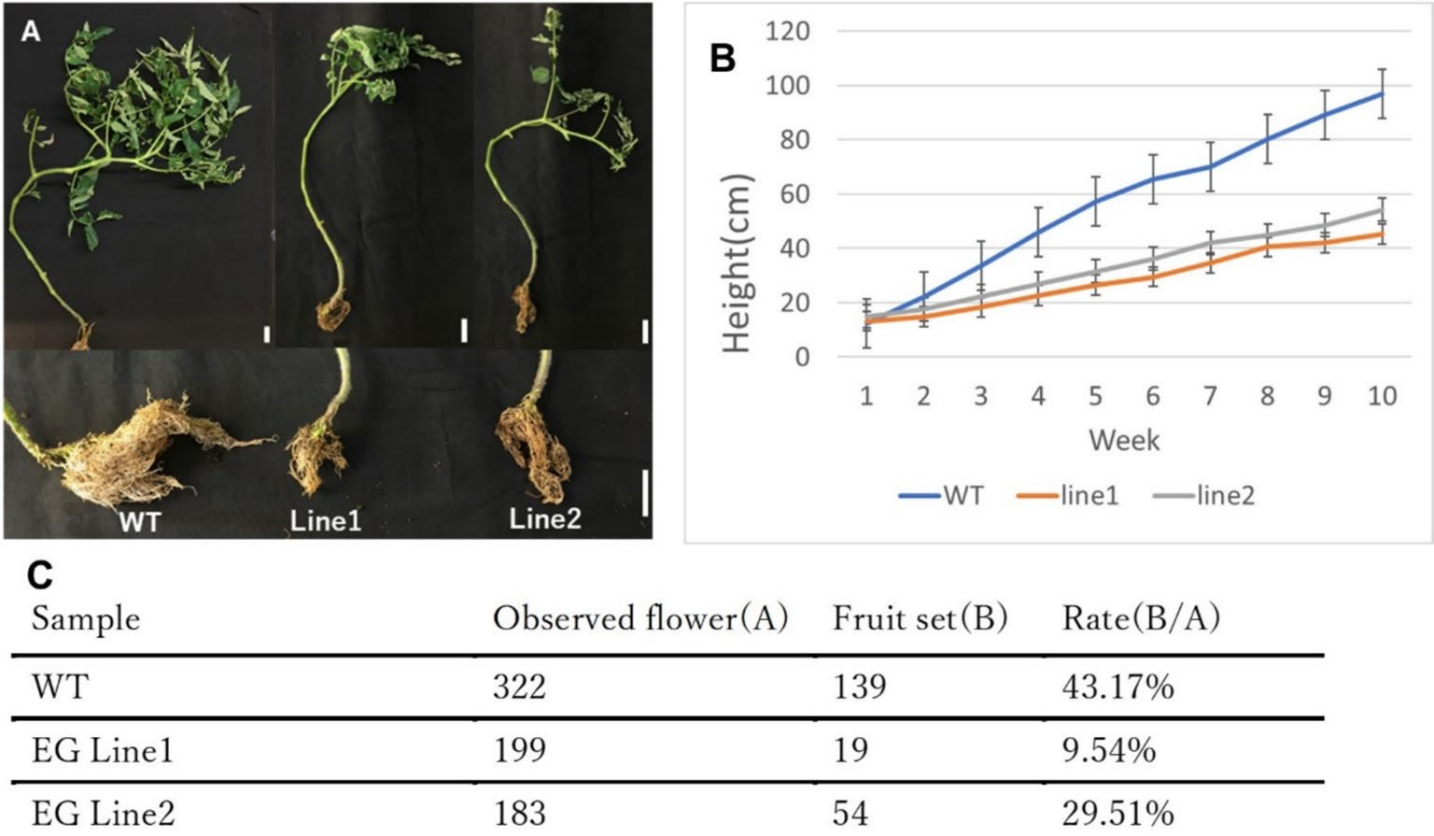
EG Lines exhibited plant growth retardation and reduced fruit set ratio. (**A**) Images are representatives of WT and EG Lines. Scale bars represent 5 cm in length. (**B**) The growing height (AVE + SE) of WT (n = 5) and EG Lines (Line 1 (n = 4), Line 2 (n = 4)) after transfer of plants from the growth room to the greenhouse. After transfer, plant height was measured every week. (**C**) Fruit set rate of WT (n = 4) and EG Lines (n = 4, each line).

A delay in growth was observed in both EG Line 1 and EG Line 2 compared to the WT (Figs. 2A, 2B). The plant heights measured 10 weeks after the start of observations are presented as mean ± standard deviation (cm): WT = 97.0 ± 4.09, EG Line 1= 45.3 ± 2.78, EG Line 2= 53.8 ± 3.20. A statistically significant difference (*p* < 0.05) was observed when comparing growth between WT and the EG lines (EG Line 1 and EG Line 2) across all weeks of measurement.

Furthermore, fruit set rates decreased significantly. Flowers and fruits were counted 21 weeks after sowing. The fruit set rate was 43.17% in the WT, whereas it was significantly lower in EG Line 1 (4.52%) and EG Line 2 (6.56%) (Fig. 2C). The decreased fruit set rates are results of abnormal flower developments, especially pollen development (Supplementary Fig. 3). The flowers in EG lines are smaller than in WT, and many flowers in EG lines are stopped developing and wilt (Supplementary Fig. 3A). The pollen in EG mutants also exhibited unshaped and small sized pollen (Supplementary Fig. 3B).

Additionally, differences in vascular structure were observed in specific regions of the stem. In the upper part of the stem (below the third mature leaf from the top) and the middle part (above the third mature leaf from the bottom), the vessel area was significantly wider in the WT compared to the EG Line 1 and EG Line 2. However, no significant differences were observed in the lower stem region (above the boundary between the stem and root) or in the leaflets (Supplementary Fig. 4).

### Differences in fruit traits in EG Line1 and EG Line2

**Fig. 3:**
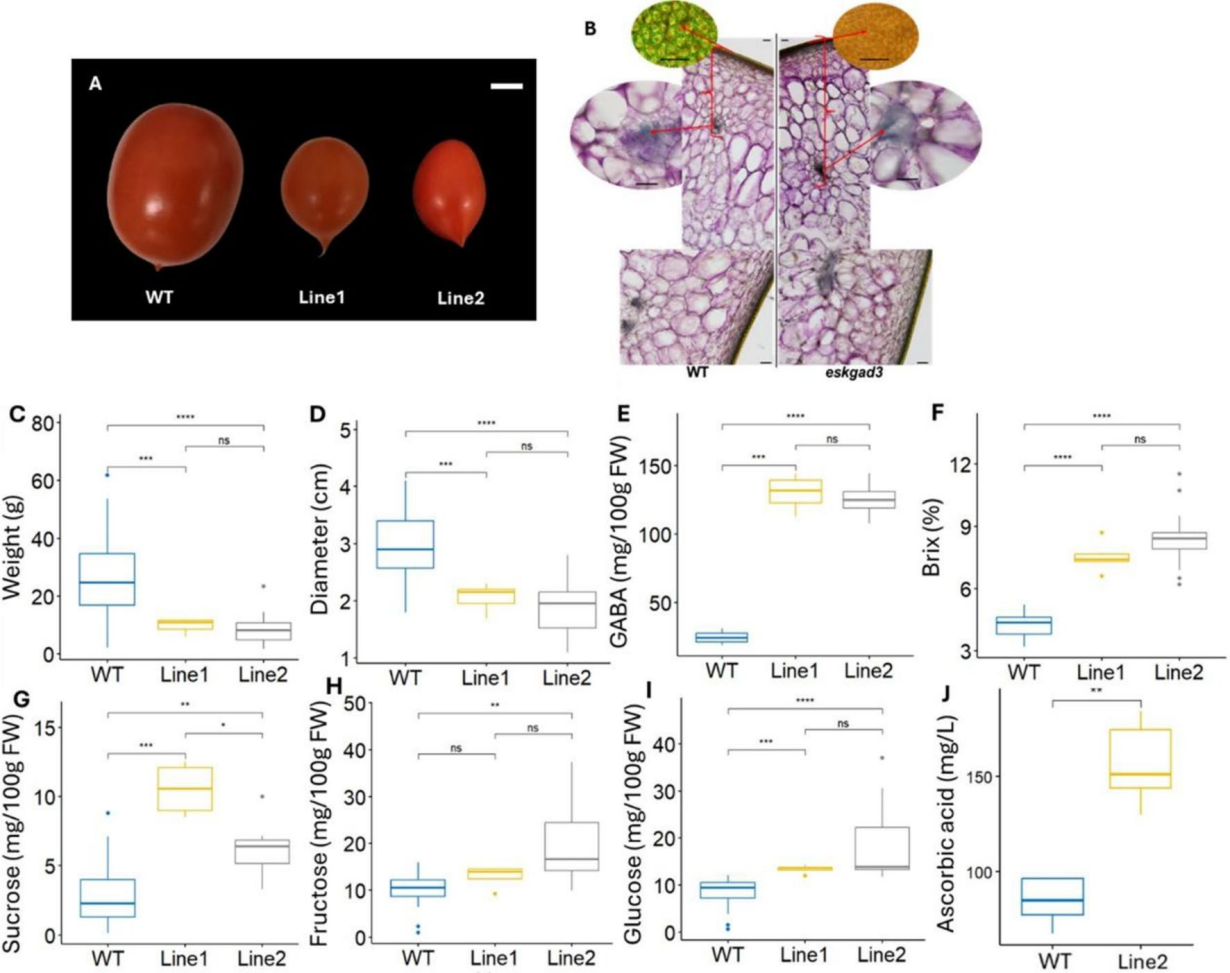
Phenotype of the red ripe tomato fruits of EG Lines. The fruits of EG Lines were smaller in size than WT, but accumulated more sugar, GABA, and ascorbic acid. (**A**) Illustrates are of representative fruits of WT and EG Lines. Scale bar represents 5 mm in length. (**B**) Xylem structure in WT and EG Lines. Scale bar is 100 μm in length. (**C**-**J**) Parameter of fruits from WT and EG Lines during spring season by using fruits’ numbers (WT, n = 15; Line 1, n = 6; Line 2, n = 15). (**C**) Fruit weight. (**D**) Fruit diameter. (**E**) GABA concentration in fruits. (**F**) Brix value of fruits. (**G**) Sucrose concentration in fruits. (H) Fructose concentration in fruits. (**I**) Glucose concentration in fruits. (**J**) Concentration of ascorbic acid in fruits. Statistical significance: ns, no significant difference; *, p < 0.05; *, p < 0.01; ***, p < 0.001; ****, p < 0.0001.

To measure fruit size, the equatorial diameter of the fruit was measured. Compared to WT, both EG Line 1 and EG Line 2 exhibited significantly smaller fruit weight and diameter, regardless of the season (Figs. 3A, 3 C, 3D; Supplementary Fig. 5A,5B).

In previous studies, the *esk1* mutant in *Arabidopsis thaliana* was reported to exhibit impaired xylem structure and secondary cell wall formation^10^. Therefore, we examined the fruit structure using microscopy. The results showed no detectable differences in xylem structure among the WT and EG lines (EG Line 1 and EG Line 2) (Fig 3B).

Regarding GABA content, a comparison of EG Line 1 and EG Line 2 with WT revealed that GABA accumulation was significantly higher in both EG Line 1 and EG Line 2 than in the WT (Fig. 3E; Supplementary Fig. 5C), regardless of the season.

For total soluble sugar content (Brix), both genome-edited lines (EG Line 1 and EG Line 2) exhibited significantly higher values compared to WT, consistently across all seasons (Fig. 3F; Supplementary Fig. 5D). To further analyze sugar composition, sucrose, fructose, and glucose levels were measured. The results indicated that sucrose and fructose levels were significantly higher in the EG Line 1 and EG Line 2 than in WT during the spring season but less significantly elevated during the summer season (Figs. 3G, 3H; Supplementary Fig. 5E,5F). In contrast, glucose content was consistently and significantly higher in the EG Line 1 and EG Line 2 across all seasons (Fig. 3I; Supplementary Fig. 5G).

From Gene Ontology (GO-) enrichment analysis and RNA-seq results, we observed the upregulation of genes associated with ascorbic acid (vitamin C) biosynthesis. Therefore, we measured ascorbic acid levels and found a significant increase in the EG Line 1 and EG Line 2 (Fig. 3J), suggesting that these plants likely enhance antioxidant production to resist and recover from stress^16^.

### EG Line1 and Line2 exhibit drought stress tolerance but doesn’t exhibit cold stress tolerance.

**Fig. 4:**
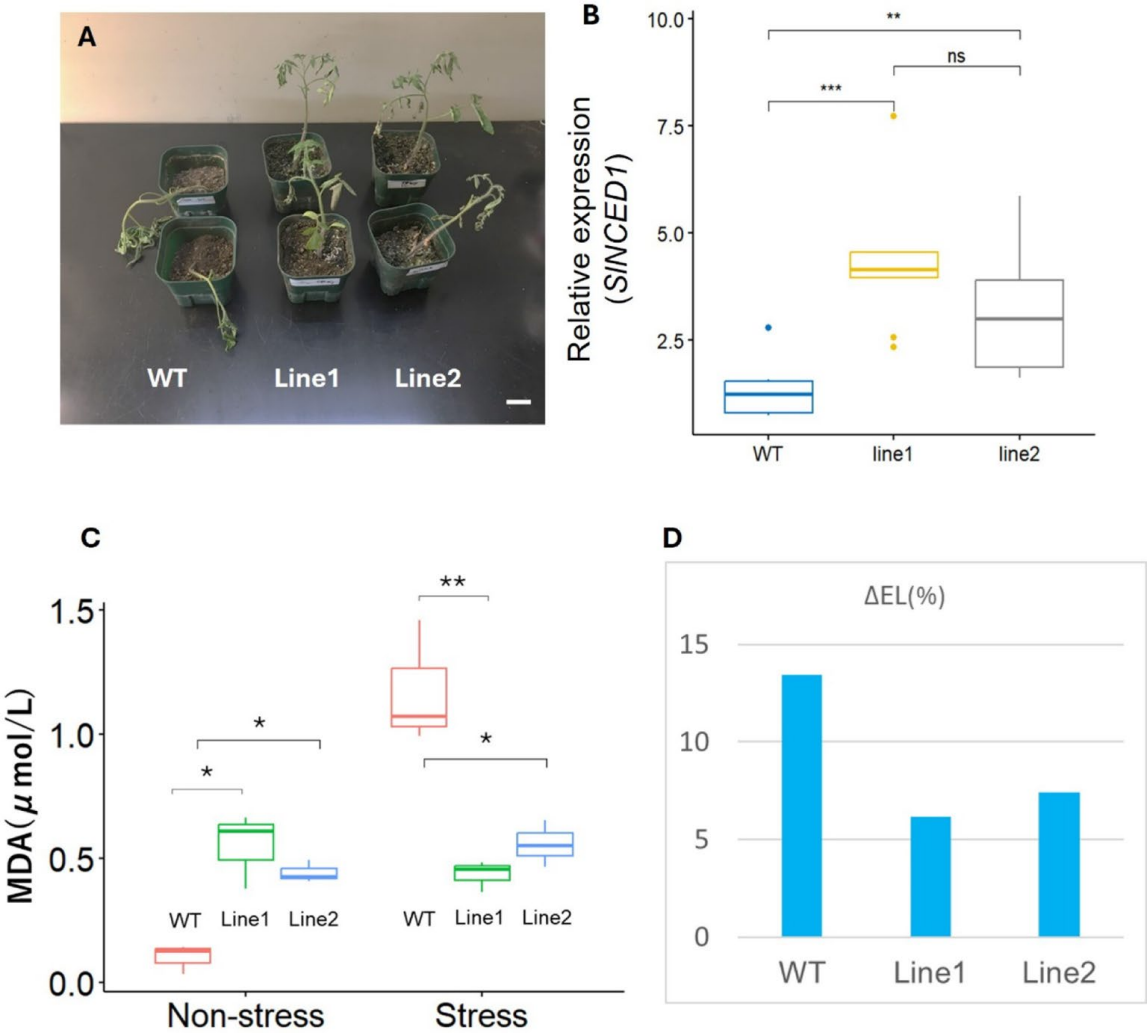
EG Lines exhibited drought stress tolerance. (**A**) Illustrates are of representative plants of WT and EG Lines 10 days after water starvation. Scale bar represents 2 cm in length. (**B**) Relative expression of *SlNCED1*. RNA was extracted from leaves of WT (n = 3) and EG Lines (n = 3, each) under normal conditions. (**C**) Concentration of MDA before stress (Non-stress) and after 10-days water starvation (Stress) (n = 3, each). (**D**) Changes of electronlyte leakage (EL) (n = 3, each). Statistical significance: ns, no significant difference; **, p < 0.01; ***, p < 0.001.

The *esk1* mutant in *Arabidopsis thaliana* has demonstrated enhanced tolerance to various abiotic stresses, including cold, drought, and salinity, which was associated with higher abscisic acid (ABA) levels^16^. To investigate whether a similar mechanism was present in the EG Line1 and EG Line2, the expression of the ABA biosynthetic gene *SlNCED1* was examined. *SlNCED1* expression was found to be upregulated in the EG Line1 and EG Line2 compared to WT under normal room temperature conditions (Fig. 4B). To assess drought stress tolerance, WT and EG lines (EG Line1 and EG Line2) were subjected to a 10-day water-starvation treatment, after which phenotypic changes, malondialdehyde (MDA) levels, and electrolyte leakage (EL) were evaluated (Figs. 4A, 4 C, 4D). Under normal conditions, MDA levels were higher in the EG Line1 and EG Line2 than in WT. However, after water-starvation, MDA levels increased significantly in WT but remained relatively unchanged in the EG Line1 and EG Line2 (Fig. 4C). Although baseline levels of EL levels were higher in the EG Line1 and EG Line2, the degree of increase in EL after drought stress was significantly greater in WT compared to the EG Line1 and EG Line2 (Fig. 4D). These results suggest that the EG Line1 and EG Line2 exhibit drought stress tolerance and sustain less cellular damage under drought conditions.

However, the EG Line1 and Line2 didn’t exhibit the cold stress tolerance (Supplementary Fig. 6A). To assess cold stress tolerance, WT and EG lines were subjected to a 7-day 4 °C cold stress environment. The MDA was evaluated 3-day and 7-day after the cold stress treatment. The MDA wasn’t significantly different between WT and EG lines after the cold stress treatment, while significantly different before the cold stress treatment (Supplementary Fig. 6B). And the expression of cold-related genes is compared between WT and EG Line 2 in room temperature condition from the RNA-seq (Supplementary Fig. 6C). *SlP5CSA* (*Solyc06g019170.3*) and *SlP5CSB* (*Solyc08g043170.4*) are orthologs of AtP5CS, and they are key genes of synthesis of proline. In the normal condition, *atesk* exhibits higher expression of proline^8^, while the EG Line 2 doesn’t exhibit significant up-regulation. And other key cold response genes (*CBF* (C-repeat binding factor) 1, *Solyc03g026290.3*; *CBF2*, *Solyc03g026270.3*; *CBF3*, *Solyc03g0260270.3*; *COLD1*, *Solyc07045330.3*; *CaM6* (Calmodulin-6), *Solyc03g098050.3*; *GT-33*, *Solyc12g043090.3*) didn’t exhibit significant changes. (Supplementary Fig. 6C)^17–20^.

### RNA-seq and GO- term- enrichment- analysis in tomato fruits

**Fig. 5:**
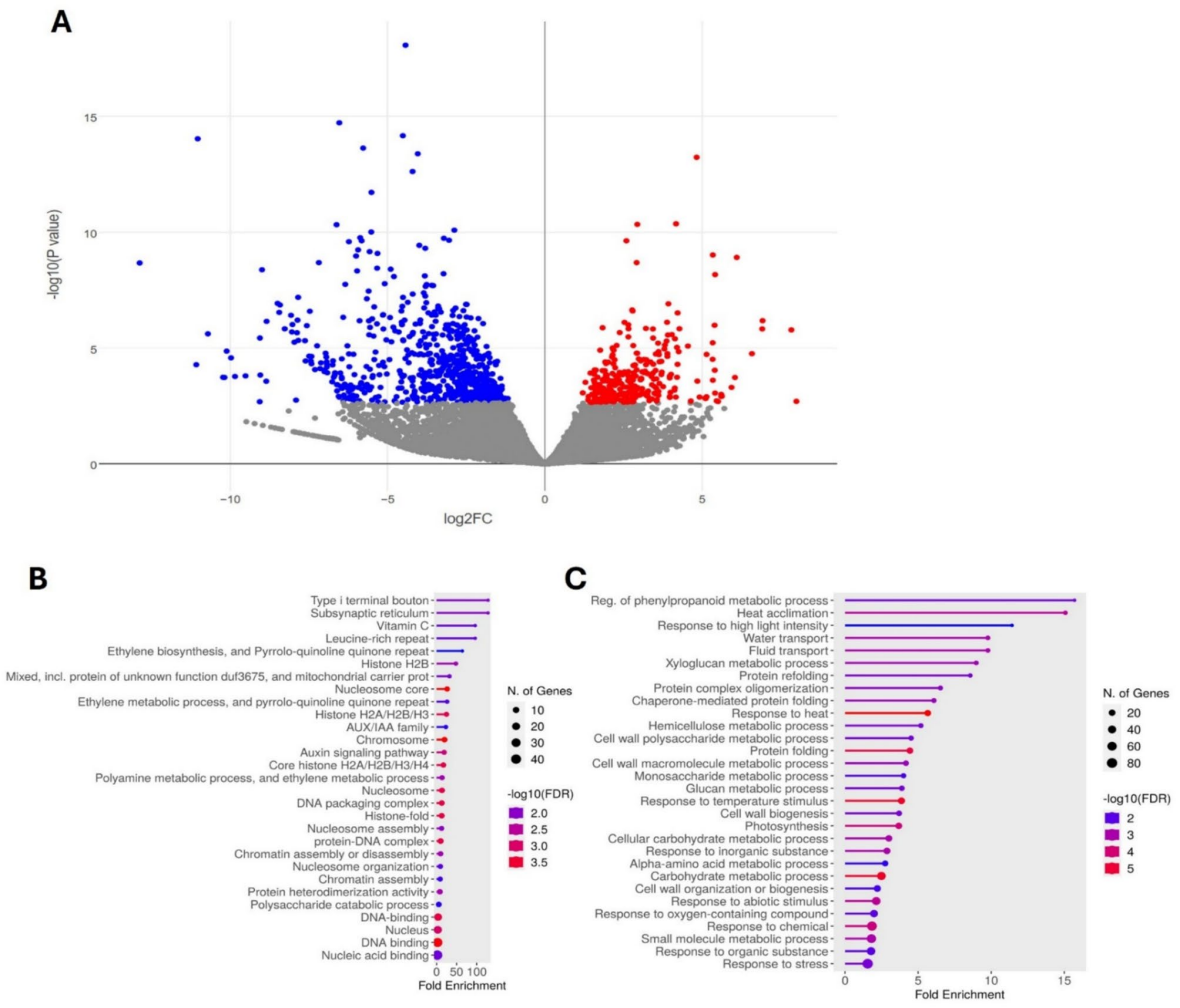
RNA-seq analysis. The red ripe tomato fruits of WT and EG Line2 were obtained and RNA was extracted from these fruits (n = 3, each). After RNA-sequenceing, gene expression was compared between WT and EG Line2. (**A**) Volcano plot. Blue dots represnet down-regulated genes, red dots represent up-regulated genes (log_2_FC > 2, padj < 0.05). Gray dots represent genes with no significant difference. (**B**, **C**) GO enrichment analysis. (**B**) Results of GO enrichment analysis for up-regulated genes. (**C**) Results of GO enrichment analysis for down-regulated genes.

Since both EG Line 1 and Line2 show decreased fruit set and difficulty of RNA extraction in ripen tomato fruits, RNA sequencing was conducted by using EG Line 2. The other studies’ results also showed significant increase in GABA content in knock out of GAD3 inhibitory domain^21–23^. RNA sequencing analysis revealed that 302 genes were significantly upregulated, while 703 genes were significantly downregulated in red ripe tomato fruits of EG Line 2 compared to WT (Fig. 5A).

Among the upregulated genes identified in the GO analysis, the most notable processes were related to vitamin C biosynthesis, the auxin signaling pathway, AUX/IAA family genes, ethylene biosynthesis, and ethylene metabolism. These biological processes play critical roles in regulating the developmental responses observed in the EG Line2. The highest upregulated gene associated with vitamin C regulation was Solyc05g054760.4 (dehydroascorbate reductase 1; *DHAR1*). Since the vitamin C biosynthesis pathway was upregulated, an increased concentration of ascorbic acid was also observed (Fig. 5B).

For sugar contents, Solyc12g008840.2 (β-galactosidase), Solyc01g111340.4 (β-xylanase) and Solyc08g081620.4 (Endo-β−1,3-glucanase precursor) were significantly upregulated (Supplementary Fig. 7). These genes have key roles in degrading polysaccharide. Moreover, Solyc09g091030.3 (β-amylase) was significantly upregulated (Supplementary Fig. 7).

For auxin transporter and regulatory genes, key upregulated genes included Solyc01g068410.4 (*SlPIN5*), Solyc10g080880.2 (*SlPIN7*), Solyc09g008175.1 (SAUR-like auxin-responsive protein family), Solyc09g065850.4 (*SlIAA3*), Solyc01g079260.4 (*SlWRKY23*), Solyc05g047460.3 (*Sl-ARF7B*), and Solyc01g103050.3 (*Sl-ARF1*). For ethylene biosynthesis and signaling, upregulated genes included Solyc01g095080.3 (*ACC2*), Solyc03g118190.4 (ethylene-responsive transcription factor), Solyc07g049530.3 (*SlACO1*), Solyc03g118190.4 (ethylene-responsive transcription factor), Solyc07g049550.3 (1-aminocyclopropane-1-carboxylate oxidase), Solyc03g044300.3 (*AP2a*) (Supplementary Fig. 7). Regarding nutrient transport and response genes, upregulated calcium-related genes included Solyc03g097100.1 (EF hand calcium-binding protein family) and Solyc01g108400.3 (calcium-dependent protein kinase-like). Additionally, the phosphate transporter Solyc02g078210.3 (*PHO2*) and Solyc01g005000.3 (*GAD3*) were identified as upregulated genes (Fig. 5B; Supplementary Fig. 7).

The GO analysis of downregulated genes identified 93 biological processes (BPs) significantly associated with key roles in plant growth and development. These BPs were classified into three main categories: stress response, cellular regulation, and transportation. A substantial number of downregulated genes in the fruits of EG Line2 were associated with stress response processes, including responses to heat and other abiotic stimuli. The significantly downregulated genes in this category included Solyc09g065660.4 (*HsfA7*), Solyc08g062960.4 (*HsfA2*), and Solyc09g009100.4 (*SolycHsfA3*), all of which are associated with heat stress response. The second category included genes involved in cellular regulation, particularly those related to cell wall biogenesis and the regulation of xyloglucan, hemicellulose, saccharide, macromolecule, and carbohydrate metabolism. The most significantly downregulated gene in this category was Solyc04g071070.2 (unknown protein, plant-type cell wall organization). The third category involved genes related to transportation, including water and lipid transport. Notable downregulated genes included Solyc01g090350.3 and Solyc01g090360.3 (non-specific lipid-transfer proteins) for lipid transport and Solyc12g044330.2 (*SlTIP2.1*), Solyc06g074820.3 (*SlTIP1.1*), and Solyc10g055630.2 (*SlPIP2.9*) for water channel activity (Fig. 5C; Supplementary Fig. 8).

## Discussion

In this study, we demonstrated that multiple desirable traits can be improved through a single genome-editing event and investigated the characteristics of the EG Line 1 and EG Line2.

Our results indicate significant differences in sugar content, GABA accumulation, and ascorbic acid level between WT and EG lines (EG Line 1 and EG Line 2) (Fig. 3). Although seasonal variations were observed, the sugar content in the red ripe fruits of the EG Line 1 and EG Line 2 was consistently higher than that of the WT. Specifically, during the spring season, the average Brix value in the EG Line 1 and EG Line 2 was approximately 1.8 times higher than in the WT. Also, the EG Line 1 and EG Line 2 of tomato produce fruits with elevated sugar content, even under a greenhouse conditions without stress induction.

Furthermore, both GABA and vitamin C contents were significantly increased in the EG Line 1 and EG Line 2, with GABA levels approximately 4 to 5 times higher and vitamin C levels approximately 1.5 times higher than those in WT (Fig. 3). These results suggest that introducing mutations into the *ESKs* and *GAD3* genes can effectively enhance the nutritional of tomato fruits. The *esks* mutants in tomato could affect the stressed phenotype and GABA content.

Growth retardation in the EG Line 1 and EG Line 2 was confirmed through analysis using T_2_ individuals. Previous research demonstrated that *esk1* mutants in *Arabidopsis thaliana* inhibit xylan acetylation, which promotes its degradation by endoxylanase and reduces secondary wall thickening^10^. This reduction in secondary wall thickening results in insufficient xylem formation, causing wilted leaves and dwarf traits. In tomatoes, it is also known that sugar content in tomato fruits increases when water availability is limited^24^. Based on these findings, it is hypothesized that the growth retardation observed in the EG Line 1 and EG Line 2 results from reduced secondary wall development caused by mutations in *SlESK* genes and that the increased fruit sugar content is attributable to decreased water transport efficiency due to defective xylem formation. Significant differences in vessel structure were observed in the stems of the top and middle regions of the EG Line 1 and EG Line 2, with a decreased vessel area compared to the WT (Supplementary Fig. 4). RNA-seq analysis revealed upregulated expression of genes related to drought-induced stress, ABA response, and water deprivation, further supporting the high sugar phenotype observed in the EG Line 2 (Supplementary Fig. 5). Additionally, the fruit set rates are significantly decreased in the EG mutants and its results are results of stunted growth in EG mutants (Supplementary Fig. 3 and Supplementary Fig. 4).

Furthermore, Solyc12g008840.2 (β-galactosidase) and Solyc08g081620.4 (Endo-β−1,3-glucanase precursor) were significantly upregulated (Supplementary Fig. 7). These genes have key roles in degrading polysaccharide in cell walls, resulting in formation of soluble sugar contents^25,26^. Moreover, Solyc09g091030.3 (β-amylase) was significantly upregulated (Supplementary Fig. 7). β-amylase is an important starch-hydrolyzing enzyme that plays a role in starch degradation into maltose and responses to environmental stress, especially drought stress^26,27^. Therefore, the increased sugar contents could result from both degradation of xylem into soluble sugars and increase in β-amylase stimulated by drought stress in fruits. Which factor is more strongly affected and how the mutants of these sugar metabolic enzymes affect the soluble sugar contents are needed to be examined in the future.

The *esk1* mutant in *Arabidopsis thaliana* has exhibited tolerance to various abiotic stresses, including drought, cold, salt and osmotic stress^8,28,29^.

In this study, the tomato EG Line 1 and EG Line 2 exhibited drought stress tolerance, as evidenced by reduced MDA and EL levels, including reduced damage under drought conditions. The observed drought tolerance suggests that the EG Line 1 and EG Line 2 may pre-emptively recognize stress signals and activate resistance mechanisms. Previous studies have reported that the *atesk1* mutants have higher ABA levels compared to WT^24^. ABA is a key phytohormone involved in regulating plant responses to abiotic stress^30^. Consistent with these findings, we also observed increased expression of *NCED1*, a key gene in ABA biosynthesis, in the EG Line 1 and EG Line 2 (Fig. 4)^24^. In contrast, the EG lines didn’t exhibit cold stress tolerance not like the *esk1* mutant in *Arabidopsis thaliana*, and direct cold response or tolerance-related genes are not upregulated (Supplementary Fig. 6). Moreover, *atesk1* mutants exhibits increased *AtCOR15* and *AtCOR78* in room temperature conditions, while tomato don’t have its orthologs or similar genes^31,32^.

In conclusion, genome editing-induced mutations in tomato *ESK1-3* and *GAD3* genes promoted sugar content, GABA levels, and vitamin C concentration, resulting in nutrient-rich tomato fruits with increased functional value. However, yield was significantly decreased because of stressed phenotype, especially in xylem formation. These trade-off results result from both degradation of xylem into soluble sugars and increase in β-amylase stimulated by drought stress in fruits. Further studies could improve soluble sugar contents in fruit with alleviated limitation of yield. Moreover, these results may help alleviative the high costs and labor-intensive processes associated with cultivating high-quality tomatoes. Producing high-sugar tomatoes typically requires significant amounts of fertilizers and labor. However, resources for agricultural production are limited and being depleted^33,34^, and the number of people engaged in farming is steadily decreasing^35,36^. In addition, the yield reduction commonly observed in the high-sugar tomato cultivation needs to be addressed in future studies.

## Materials and methods

### Plant and growth conditions

Tomato (*Solanum lycopersicum*) plants used in this study were provided by Sanatech Life Science Co., Ltd. The cultivation room was maintained at a constant temperature of 25 °C, with a photoperiod of 16 h of light and 8 h of darkness. In the greenhouse, the temperature ranged from 11 °C to 40 °C during the spring season and from 20 °C to 60 °C during the summer season. Fertilizers included Otsuka House No. 1 and Otsuka House No. 2 (OAT Agrio Co., Ltd.).

### Construction of vector plasmid

To create the pPcUfcoCas9-SlESKGAD3 plasmid, a reaction mixture containing 6 μl of CutSmart buffer, 2 μl of the restriction enzymes *ApaI* and *AscI*, and 50 μl of the plasmid sample pPcUbi was incubated at 37 °C for 60 min. Subsequently, amplicons listed in Supplementary Table 5 (#1-#4) were amplified using KOD-Plus Neo (TOYOBO) and ligated with the digested pPcUbi plasmid.

To construct pPcUfcoCas9-SlESK1,2GAD3 (Fig. 1), a reaction mixture containing 6 μl of CutSmart buffer, 2 μl of the restriction enzyme *Xma*l, and 48 μl of the pPcUfcoCas9-SlESKGAD3 plasmid sample was prepared, and the reaction was performed under the same conditions. The target sites were amplified and inserted into the restricted pPcUfcoCas9-SlESKGAD3 plasmid (#5-#6).

For plasmid purification, 150 μl of Milli-Q water was added to the restriction enzyme-treated plasmid sample on ice. Then, 200 μl of the lower layer of Tris-saturated phenol- chloroform-isoamyl alcohol was added, thoroughly mixed, and centrifuged at 15,300 × g, 4 °C for 5 min. The supernatant was collected, and 40 μl of 3 M sodium acetate was added, followed by thorough mixing. Next, 400 μl of isopropanol was added, mixed, and centrifuged at 15,300 × g at 4 °C for 20 min. The supernatant was discarded, and the pellet was washed with 500 μl of 70% ethanol, inverted five times, and centrifuged again at 15,300 × g at 4 °C for 5 min.

KOD-Plus Neo (TOYOBO) was used to amplify the genome-editing constructs for each target site: *SlGAD3*-target, *SlESK*-target1 and *SlESK*-target2 by PCR. The reaction composition is listed in Supplementary Table 5 (#1-#6), and the PCR conditions were allowed according to the manufacturer’s instructions. PCR products were extracted from agarose gel using the QIAquick Gel Extraction Kit (QIAGEN).

The ligation reaction was performed using the In-Fusion Snap Assembly Master Mix (Takara Bio). The reaction tube was kept on ice, and 25 μl of Stellar Competent Cells (Takara Bio) were added. Heat shock was applied at 42 °C for 45 s to transform the plasmid into *E. coli*. Subsequently, 100 μl of SOC medium was added, and the culture was incubated with shaking at 180 rpm at 37 °C for 60 min. The mixture was then spread on LB plates containing 50 μg/ml kanamycin and cultured at 37 °C overnight. The desired vector was confirmed by sequencing the plasmid samples verify the correct insertion.

### Tomato transformation using the agrobacterium method

Tomato transformation was performed following the high-efficiency transformation protocol^37^. Both the prepared vector plasmid and the Super-Agrobacterium plasmid pBBRacdSgadTAmp^38^ were transformed into *Agrobacterium tumefaciens* strain GV2260. After infection of *Agrobacterium* into tomato cotyledons, plants were regenerated as described previously^37^.

### Preparation of genomic DNA and amplification of target DNA regions

About 5 mm^2^ section of a true leaf from each plant was placed in a 1.5 ml tube and frozen with liquid nitrogen. Next, 100 μl of Buffer A (1 M Tris–HCl [pH 9.5] 30 ml, KCl 23 g, 0.5 M EDTA 6 ml, distilled water to a final volume of 300 ml) was added, and the leaf section was homogenized using a hand pestle. The tube was then incubated at 95 °C for 5 min. After incubation, the tube was cooled on ice and centrifuged at 17,600 × g 4 °C for 5 min. The resulting supernatant was transferred to a new tube.

KOD-FX Neo (TOYOBO) was used for the PCR amplification of the target DNA regions. The primers used for MultiNA sample preparation were as follows: *SlGAD3-multiF* and *SlGAD3-multiR* for the *SlGAD3* target region, *ESK1-target2-multinaF* and *ESK1-target2-multinaR* for the *ESK1* target region, *ESK2-target2-multinaF* and *ESK2-target2-multinaR* for the *ESK2* target region, and *ESK3-target2-multinaF* and *ESK3-target2-multinaR* for the *ESK3* target region (Supplementary Table 6, #1-#7). After PCR amplification, the sizes of the PCR products were confirmed by agarose gel electrophoresis.

The MultiNA system (SHIMADZU) was used to identify candidate individuals with genome-editing-induced mutations. First, 4 μl of the PCR products from the target gene regions were incubated at 95 °C for 5 min and allowed to anneal at room temperature. Size ladder preparation was performed according to the MultiNA operating protocol. When a different band pattern was detected by the MultiNA system, the PCR products were cloned into the pGEM-T *Easy* vector (Promega) and transformed into *E. coli*. After colony formation, PCR was performed using the primers M13-47 and RV-P (Supplementary Table 6, #8).

The resulting PCR product was treated with Illustra ExoProStar (Cytiva) according to the manufacturer’s instructions. Finally, the samples were sequenced using the primer M13-47.

###  Cultivation of T_1_ and T_2_ generations

A 1% hydrochloric acid solution was poured into a beaker, fully submerging the seeds extracted from fully ripe red tomatoes. The seeds were stirred in the solution for 15 min. They were then thoroughly rinsed with water and dried.

Seeds collected from individuals with confirmed mutations, along with WT seeds for comparison, were directly sown on rock wool. After root establishment, genomic DNA was extracted from the leaves of the plants to confirm the inheritance of mutations.

### Agrobacterium persistence test

Approximately 5 mm^2^ segments of plant leaves were placed in a sterile tube and homogenized using a pestle in 200 μl of sterile water. The homogenized solution was briefly centrifuged for approximately 10 s. Then, 50 μl of the supernatant was streaked onto AB medium (K₂HPO₄ 600 mg, NaH₂PO₄ 200 mg, NH₄Cl 200 mg, KCl 30 mg, CaCl₂·2H₂O 20 ml, FeSO₄·7H₂O (2.5 mg/ml) 20 ml, Agarose 3 g, 5% Glucose 20 ml, MgSO₄·7H₂O (1 M) 240 μl, Kanamycin (50 mg/ml) 200 μl, distilled water 200 ml), and incubated at 28 °C for 5 days. As controls, streaking and culturing were also performed on the same medium using *Agrobacterium tumefaciens* GV2260 harboring the plasmid pPcUfcoCas9-SlESKGAD3 as a positive control and sterile water as a negative control.

### Evaluation of exogenous gene persistence and off-target mutations

An exogenous gene residual test was performed using KOD-FX Neo (TOYOBO). The primers listed in Supplementary Table 6 (#9-#19) were used to amplify the entire plasmid region of *pPcUfcocas9-SlESKGAD3*. Additionally, PCR was conducted under identical conditions using pPcUfcoCas9-SlESKGAD3 as a positive control and genomic DNA extracted from WT as a negative control.

Off-target mutations were identified using Cas-OFFinder^39^. The following parameters were applied: PAM type, 5’-NGG-3’; target-genome, *Solanum lycopersicum* (SL4.0); maximum mismatch number, 2; DNA bulge size, 1; RNA bulge size, 1. Potential off-target mutation sites located at the target loci were selected. Among these, only sites where gene exons were registered in the *Solanum lycopersicum* (SL4.0) genome database were chosen for confirmation. To amplify candidate off-target mutation sites, PCR was performed using KOD-FX Neo (TOYOBO) with primers listed in Supplementary Table 6 (#20-#28). After amplification, the PCR products were sequenced for mutation verfication.

### Fruit measurement and analysis

#### Fruit size and weight

Fruit size was determined by measuring the maximum diameter of the fruit along the equatorial plane using a vernier caliper. Fruit weight was measured using an electronic balance.

#### Brix measurement

The Brix value was measured using a digital refractometer (AS ONE).

#### GABA content measurement

GABA content was measured using the GABA Miel kit (Enzyme Sensor Co., Ltd.) according to the manufacturer’s instructions. Briefly, distilled water equivalent to nine times the fruit’s weight was added to the fruit sample, which was then crushed using a mortar. The resulting mixture was filtered, and 50 μl of the filtrate, along with the reaction standard solution, was placed in a measurement cell at room temperature. Subsequently, 500 μl of Solution A was added, and the reaction was allowed to proceed for 10 min. Then, 500 μl of Solution B was added, and the mixture was incubated for another 10 min. The GABA content (mg/100 g fresh weight) was determined using an LED colorimeter (445 nm) calibrated with distilled water and the reaction standard solution.

#### Plant height measurement

Seeds were sown and seedlings were grown in the cultivation room until they reached approximately 40 cm in height. The plants were then transferred to a greenhouse, where plant height was measured weekly. Measurements were taken from the aerial part of the plant.

### Statistical analysis

Statistical analysis was performed using R software (R Core Team, 2023). The Kruskal–Wallis test was used to compare three or more groups with different sample sizes. The Wilcoxon signed-rank test was applied to account for data asymmetry and other non-parametric characteristics, allowing for pairwise comparisons. Additionally, the Mann–Whitney U test was performed, with correction for multiple comparisons using the Holm method.

For statistical analysis of plant height, the Wilcoxon signed-rank test (equivalent to the Mann–Whitney U test) was used because homoscedasticity could not be assumed, as evaluated using the F-test and Levene’s test. Plant growth was calculated by subtracting the previous week’s height from the current week’s height, and cumulative growth values were compared.

### MDA measurement

To measure MDA levels, 4-week-old plants were used. Prior to drought stress treatment, 100 mg of fresh leaves were collected and immediately frozen in liquid nitrogen. Under drought stress conditions, both WT and EG lines (EG Line 1 and EG Line 2) were subjected to a 10-day water starvation period, after which leaves were sampled for the same manner as the non-stressed condition. The collected leaves were finely ground in 1.5 ml of a 10% (v/v) solution of trichloroacetic acid (TCA) solution. Following centrifugation at 17,600 × g for 15 min, 1 ml of the resulting supernatant was mixed with 1 ml of a 0.6% (w/v) thiobarbituric acid (TBA) solution prepared in 10% TCA. The mixture was heated in boiling water for 20 min and then cooled to room temperature. Absorbance was measured at 450 nm, 532 nm, and 600 nm using a DU-800 spectrophotometer (Beckman Coulter). The MDA concentration was calculated using the following formula: MDA(μmol/L) = [6.45 × (A_532_-A_600_) – 0.56 × A_450_].

The MDA of the cold stress treatment also measured, after 3-days and 7-days cold stress treatment at a 4 °C incubator.

### EL measurement

Fresh leaves from each 4-week-old plant were rinsed thoroughly with Milli-Q water (MQ) and placed in tubes filled with MQ to ensure full submersion of the leaf tissue. The tubes were maintained at room temperature for 24 h, after which EL was measured as C_1_ using an ion conductivity meter (Lutron).

Next, the samples were subjected to high-pressure sterilization at 121 °C for 20 min and allowed to cool to room temperature. EL measurements were then recorded as C_2_. The percentage of EL was calculated using the following formula: EL (%) = (C_1_/C_2_) × 100.

### RNA extraction and purification

For RNA extraction, 100 mg of fresh leaves or red ripe fruits harvested from June to July were frozen in liquid nitrogen and finely ground. Total RNA was extracted using TRIzol (Thermo Fisher Scientific) according to the manufacturer’s instructions. A total of 2 μg of RNA was used for complementary DNA (cDNA) synthesis using the High-Capacity cDNA Reverse Transcription Kit (Thermo Fisher Scientific). The primers *SlNCED1*-F and *SlNCED1*-R were used to amplify *SlNCED1* for real-time PCR analysis (Supplementary Table 6, #29). Real-time PCR and relative abundance calculations were performed as described previously (Abellatif et al., 2002). The *SlEXPRESSED* gene was used as an endogenous control for gene expression analysis (Choi et al., 2018 Plant Biotechnol).

### RNA-seq analysis

RNA sequencing analysis was outsourced to Rhelixa Co., Ltd. Quality control (QC) score were assessed using FastQC software (Version 0.11.7; https://www.bioinformatics.babraham.ac.uk/projects/fastqc/). Low-quality bases (Q < 20) and adapter sequences were trimmed using Trimmomatic software (Version 0.38) with the following parameters: ILLUMINACLIP: path/to/adapter.fa:2:30:10 LEADING:20 TRAILING:20 SLIDINGWINDOW:4:15 MINLEN:36. The trimmed reads were aligned to the reference genome using the RNA-seq aligner HISAT2 (Version 2.1.0). The HISAT2-generated.sam files were converted into.bam files using Samtools (Version 1.9). The resulting.bam files were used to estimate the abundance of uniquely mapped reads with featureCounts (Version 1.6.3). Raw read counts were normalized using transcripts per million (TPM). The samples were clustered using the Wald method based on the Euclidean distances of the normalized counts, employing stats (Version 3.6.1) and gplots (Version 3.0.1.1) R packages. Principal component analysis (PCA) was performed on the normalized counts, and each sample was projected onto two-dimensional plane based on the first and second PCA axes using the same R packages. Pearsonʼs correlation coefficients of the normalized counts were calculated to assess correlation between samples. Histograms and pair plots of the normalized counts were generated using the stats and gplots R packages. Heatmaps were created from Z-scores of the normalized counts using the same R packages. Raw read counts were further normalized using relative log expression normalization, and differential expression analysis was conducted with DESeq2 (Version 1.24.0). Differentially expressed genes (DEGs) were identified using the thresholds of |log_2_ fold change(FC) |> 1 and adjusted p-value < 0.05 (and < 0.1), calculated using the Benjamini-Hochberg (BH) method for multiple testing correction.

GO enrichment analysis was performed using ShinyGO 0.80 (http://bioinformatics.sdstate.edu/go/). The *Slycopersicum*_eg_gene Ensembl ID was employed, with the *Solanum lycopersicum* SL3.0 gene annotation used as the database. The false discovery rate (FDR) cutoff was set to 0.05.

### Ascorbic acid measurement

Tomato fruit (1.0 g) was homogenized using a mortar and pestle and mixed with 2.0 ml of 5% (w/v) metaphosphoric acid. After centrifugation at 12,000 × g for 3 min, the supernatant was collected as a crude extract. The total ascorbic acid content was measured using the Ascorbic Acid Test Kit (Merck, Darmstadt, Germany) with the RQ Flex Plus 10 analyzer (Merck, Darmstadt, Germany).

### Microscopy analysis

Microscopy analysis of stems, flowers and fruits was performed by preparing tissue sections using a Vibrating-Blade Microtome VT1200 (Leica) and staining with Toluidine Blue for xylem and with Iodine potassium for pollen. Observations were conducted using a BX50 microscope (Olympus).

## Supplementary Information

Supplementary Information 1.

Supplementary Information 2.

## Data Availability

The transcriptome data was used Solanum lycopersicum SL3.0 gene annotation and is available in the NCBI database: the accession number is GCF_000188115.4. The sequence data used in this study can be found in the GenBank data libraries under the following accession numbers: SlESK1 (Solyc03g096030), SlESK2 (Solyc06g051350), SlESK3 (Solyc05g052540), SlGAD3 (Solyc01g005000). And the data presented in this study are available on request from the corresponding author.
