# Supplementary material for: Enhancing tomato quality, high sugar content and GABA accumulation, with mutations in ESKs and GAD3 genes
Source: Sci Rep. 2025 Dec 2;16:161. doi: 10.1038/s41598-025-28888-5 (PMC12764964; doi:10.1038/s41598-025-28888-5)
Supplement: Supplementary file 1 — Supplementary Information 1. [file 41598_2025_28888_MOESM1_ESM.docx]

**Supplementary file**

**Supplementary Table 1. Sequences of *eskgad3* lines in T_0_ generations.**

| line | Sample name | Target gene | Sequence | Mutation rate |
| --- | --- | --- | --- | --- |
| *esksgad3*  *esksgad3*  *esksgad3* | 58 | *GAD3* | CCCGAATGCCAAA---GTGG CCCGAATGCC---AAAGTGG | 1/4  1/4 |
|  |  | *ESK1*target | AGAG----TTCTTTACCCAA AGA----GTTCTTTACCCAA  AGA-----TTCTTTACCCAA | 1/4  1/4  2/4 |
|  |  | *ESK2*target1 | AGAAGACTGTTCTTTACCCAA AGA------TCTTTACCCAA | 3/4  1/4 |
|  |  | *ESK3*target1 | AGAAGACTGTTCTTTACCCAA AGA------TCTTTACCCAA | 2/4  2/4 |
|  |  | *ESK1*target2 | ATGCCACTGTGGA----TAC | 3/4 |
|  | 60 | *GAD3* | CCCGAATGCCAAAAAAGATGG | 1/4 |
|  |  | *ESK1*target1 | AGA—CTGTTCTTTACCCAA  --AGACTGTTCTTTACCCAA | 1/3  2/3 |
|  |  | *ESK2*target1 | AGA------TCTTTACCCAA | 4/4 |
|  |  | *ESK3*target1 | AG----TGTTCTTTACCCAA AGAG----TTCTTTACCCAA AGA----GTTCTTTACCCAA | 2/4  1/4  1/4 |
|  |  | *ESK1*target2 | 「ATGCCACTG-----------」-- ATGCCACT---------TAC  ATGCC------------AC | 2/4  1/4  1/4 |
|  | 69 | *GAD3* | CCCGAATGCCAAAAAAGATGG CCCGAATGCCAAAAAAGTTGG | 1/4  2/4 |
|  |  | *ESK1*target1 | AGATGACTGTTCTTTACCCAA | 3/4 |
|  |  | *ESK2*target1 | AGAAGACTGTTCTTTACCCAA | 1/3 |
|  |  | *ESK3*target1 | AGAAGACTGTTCTTTACCCAA AGA------TCTTTACCCAA | 3/6  2/6 |
|  |  | *ESK1*target2 | ATGCCACTGTGGA----TAC | 4/4 |
|  | 76 | *ESK1*target1 | AGATGACTGTTCTTTACCCAA | 3/4 |
|  |  | *ESK2*target1 | AGAAGACTGTTCTTTACCCAA AGA—CTGTTCTTTACCCAA  --AGACTGTTCTTTACCCAA | 2/4  1/4  1/4 |
|  |  | *ESK3*target1 | AGAAGACTGTTCTTTACCCAA  ---------「--AGACTGTTCTTTACCCAA」  -------「--AGACTGTTCTTTACCCAA」 | 2/4  1/4  1/4 |
|  |  | *ESK1*target2 | ATGCCACTGT-------TAC ATGCCA-------ATTTTAC | 1/4  1/4 |
|  | 91 | *GAD3* | CCCGAATGCCAA-----TGG | 1/3 |
|  |  | *ESK1*target1 | AGATGACTGTTCTTTACCCAA | 1/4 |
|  |  | *ESK2*target1 | AGAAGACTGTTCTTTACCCAA  -CAGACTGTTCTTTACCCAA | 1/4  3/4 |
|  |  | *ESK3*target1 | AGA-ACTGTTCTTTACCCAA | 4/4 |
|  |  | *ESK1*target2 | 「ATGCCACTGTGG--------」--GGC | 4/4 |
|  | 102 | *GAD3* | CCCGAATGCCAAAAAAGGTGG | 2/3 |
|  |  | *ESK1*target1 | AGA----------TACCCAA | 4/4 |
|  |  | *ESK2*target1 | AGA----------TACCCAA  A-----TATTCTTTACCCAA  AGA-----TTCTTTACCCAA | 2/4  1/4  1/4 |
|  |  | *ESK3*target1 | AGAG----TTCTTTACCCAA | 4/4 |
|  |  | *ESK1*target2 | ATGCCACTGTGGAAT---AC | 4/4 |
|  | 105 | *ESK1*target1 | AGA-----TTCTTTACCCAA | 1/2 |
|  |  | *ESK2*target1 | AGA----GTTCTTTACCCAA  AGA--------------CAA  AGAG----TTCTTTACCCAA | 1/4  2/4  1/4 |
|  |  | *ESK3*target1 | AGA-----------ACCCAA  AGA--CTGTTCTTTACCCAA  AGA--CGGTTCTTTACCCAA | 2/4  1/4  1/4 |
|  | 127 | *GAD3* | CCCGAATGCCAAA----TGG CCCGAATGCCAAAAAAGTTGG CCCGAATGCCAAAAGAGTTGG | 2/4  1/4  1/4 |
|  |  | *ESK1*target1 | AGA-----TTCTTTACCCAA  AGA-----TTCTGTACCCAA | 3/4  1/4 |
|  |  | *ESK2*target1 | AGAAGACTGTTCTTTACCCAA AGA------TCTTTACCCAA | 2/4  2/4 |
|  |  | *ESK3*target1 | AGA-----TTCTTTACCCAA | 4/4 |
|  | 150 | *ESK1*target1 | AGA--CTGTTCTTTACCCAA | 4/4 |
|  |  | *ESK2*target1 | AGA----GTTCTTTACCCAA AGAG----TTCTTTACCCAA | 1/2  1/2 |
|  |  | *ESK3*target1 | AGA--CTGTTCTTTACCCAA | 2/2 |
|  |  | *ESK1*target2 | ATGCCACTGTGGAAT---AC | 3/4 |
|  | 152 | *ESK2*target1 | --AGACTGTTCTTTACCCAA | 1/2 |

**Supplementary Table 2. Sequences of *eskgad3* lines in T_1_ generations.**

| line | Sample name | Target gene | Sequence |
| --- | --- | --- | --- |
| *esksgad3* | 76-1 | *ESK1*target1 | AGATGACTGTTCTTTACCCAA |
|  |  | *ESK2*target1 | --AGACTGTTCTTTACCCAA |
|  |  | *ESK3*target1 | hetero |
|  | 76-2 | *ESK1*target1 | AGATGACTGTTCTTTACCCAA |
|  |  | *ESK2*target1 | hetero |
|  |  | *ESK3*target1 | hetero |
|  |  | *ESK1*target2 | hetero |
|  | 76-3 | *ESK1*target1 | AGATGACTGTTCTTTACCCAA |
|  |  | *ESK2*target1 | AGAAGACTGTTCTTTACCCAA |
|  |  | *ESK3*target1 | ---------「--GACTGTTCTTTACCCAA」 |
|  |  | *ESK1*target2 | ATGCCACTGTG-------TTAC |
|  | 76-4 | *ESK1*target1 | AGATGACTGTTCTTTACCCAA |
|  |  | *ESK2*target1 | --AGACTGTTCTTTACCCAA |
|  |  | *ESK3*target1 | AGAAGACTGTTCTTTACCCAA |
|  |  | *ESK1*target2 | ATGCCACTGTGGA----TAC |
|  | 76-5 | *ESK1*target1 | AGATGACTGTTCTTTACCCAA |
|  |  | *ESK2*target1 | hetero |
|  |  | *ESK3*target1 | hetero |
|  |  | *ESK1*target2 | hetero |
| *esksgad3* | 76-6 | *ESK1*target1 | AGATGACTGTTCTTTACCCAA |
|  |  | *ESK2*target1 | --AGACTGTTCTTTACCCAA |
|  |  | *ESK3*target1 | ---------「--GACTGTTCTTTACCCAA」 |
|  |  | *ESK1*target2 | ATGCCACTG-------TTAC |
|  | 76-7 | *ESK1*target1 | AGATGACTGTTCTTTACCCAA |
|  |  | *ESK2*target1 | --AGACTGTTCTTTACCCAA |
|  |  | *ESK3*target1 | AGAGACTGTTCTTTACCCAA |
|  |  | *ESK1*target2 | hetero |
|  | 76-8 | *ESK1*target1 | AGATGACTGTTCTTTACCCAA |
|  |  | *ESK2*target1 | hetero |
|  |  | *ESK3*target1 | AGAGACTGTTCTTTACCCAA |
|  |  | *ESK1*target2 | ATGCCACTG-------TTAC |
|  | 76-9 | *ESK1*target1 | AGATGACTGTTCTTTACCCAA |
|  |  | *ESK2*target1 | AGAAGACTGTTCTTTACCCAA |
|  |  | *ESK3*target1 | ---------「--GACTGTTCTTAACACTG」 |
|  |  | *ESK1*target2 | hetero |
|  | 76-10 | *ESK1*target1 | AGATGACTGTTCTTTACCCAA |
|  |  | *ESK2*target1 | hetero |
|  |  | *ESK3*target1 | hetero |
|  | 76-11 | *ESK1*target1 | AGATGACTGTTCTTTACCCAA |
|  |  | *ESK2*target1 | hetero |
|  |  | *ESK3*target1 | TTAGACTGT-----------」--- |
|  |  | *ESK1*target2 | ATGCCACTG-------TTAC |
|  | 102-6 | *ESK3*target1 | hetero |
|  | 102-10 | *ESK2*target1 | hetero |
|  | 105-1 | *ESK1*target1 | hetero |
|  | 105-2 | *ESK1*target1 | hetero |
|  |  | *ESK3*target1 | AGA--C---------CCCAA |
|  | 105-3 | *ESK1*target1 | AGA-----TTCTTTACCCAA |
|  |  | *ESK2*target1 | hetero |
|  |  | *ESK3*target1 | --AGACTGTTCTTTACCCAA |
|  | 105-4 | *ESK1*target1 | hetero |
|  |  | *ESK2*target1 | hetero |
|  |  | *ESK3*target1 | --AGACTGTTCTTTACCCAA |
|  | 105-5 | *ESK1*target1 | AGATAC-GCTCT--------」-TA |
|  |  | *ESK2*target1 | hetero |
|  |  | *ESK3*target1 | AGA--C---------CCCAC |
| *esksgad3* | 105-6 | *ESK1*target1 | hetero |
|  |  | *ESK2*target1 | AGA----GTTCTTTACCCAA |
|  |  | *ESK3*target1 | AGA--CT-TT-T---CCCAA |
|  | 105-7 | *ESK1*target1 | AGA-----TTCTGTACCCAA |
|  |  | *ESK2*target1 | hetero |
|  |  | *ESK3*target1 | hetero |
|  | 105-8 | *ESK1*target1 | AGA-----TTCTGTACCCAA |
|  |  | *ESK2*target1 | hetero |
|  |  | *ESK3*target1 | hetero |
|  | 127-1 | *GAD3* | CCCGAATGCCAAAAAAGTTGG |
|  |  | *ESK1*target1 | AGA-----TTCTTTACCCAA |
|  |  | *ESK2*target1 | hetero |
|  |  | *ESK3*target1 | AGA-----TTCTTTACCCAA |
|  | 127-2 | *GAD3* | CCCGAATGCCAAA----TGG |
|  |  | *ESK1*target1 | AGA-----TTCTTTACCCAA |
|  |  | *ESK2*target1 | mono |
|  |  | *ESK3*target1 | AGA-----TTCTTTACCCAA |
|  | 127-3 | *GAD3* | CCCGAATGCCAAA----TGG |
|  |  | *ESK2*target1 | AGAAGACTGTTCTTTACCCAA |
|  |  | *ESK3*target1 | AGA-----TTCTTTACCCAA |
|  | 127-4 | *GAD3* | CCCGAATGCCAAAAAAGTTGG |
|  |  | *ESK1*target1 | AGA-----TTCTTTACCCAA |
|  |  | *ESK2*target1 | hetero |
|  |  | *ESK3*target1 | AGA-----TTCTTTACCCAA |
|  | 127-5  (EG Line1) | *GAD3* | CCCGAATGCCAAA----TGG |
|  |  | *ESK1*target1 | AGA-----TTCTTTACCCAA |
|  |  | *ESK2*target1 | AGA------TCTTTACCCAA |
|  |  | *ESK3*target1 | AGA-----TTCTTTACCCAA |
|  | 127-6 | *GAD3* | CCCGAATGCCAAA----TGG |
|  |  | *ESK1*target1 | AGA-----TTCTTTACCCAA |
|  |  | *ESK2*target1 | hetero |
|  |  | *ESK3*target1 | AGA-----TTCTTTACCCAA |
|  | 127-7 | *GAD3* | CCCGAATGCCAAA----TGG |
|  |  | *ESK1*target1 | AGA-----TTCTTTACCCAA |
|  |  | *ESK2*target1 | AGAAGACTGTTCTTTACCCAA |
|  |  | *ESK3*target1 | AGA-----TTCTTTACCCAA |
| *esksgad3* | 127-8 | *GAD3* | hetero |
|  |  | *ESK1*target1 | AGA-----TTCTTTACCCAA |
|  |  | *ESK2*target1 | AGA------TCTTTACCCAA |
|  |  | *ESK3*target1 | AGA-----TTCTTTACCCAA |
|  |  | *ESK1*target2 | hetero |
|  | 127-9  (EG Line2) | *GAD3* | CCCGAATGCCAAAAAAGTTGG |
|  |  | *ESK1*target1 | AGA-----TTCTTTACCCAA |
|  |  | *ESK2*target1 | hetero |
|  |  | *ESK3*target1 | AGA-----TTCTTTACCCAA |
|  | 127-10 | *GAD3* | CCCGAATGCCAAA----TGG |
|  |  | *ESK1*target1 | AGA-----TTCTTTACCCAA |
|  |  | *ESK3*target1 | AGA-----TTCTTTACCCAA |
|  | 150-1 | *ESK1*target1 | hetero |
|  |  | *ESK2*target1 | AGAGA----TCTTTACCCAA |
|  |  | *ESK3*target1 | AGA----GTTCTTTACCCAA |
|  |  | *ESK1*target2 | hetero |
|  | 150-2 | *ESK1*target1 | hetero |
|  |  | *ESK2*target1 | AGA----GTTCTTTACCCAA |
|  |  | *ESK3*target1 | hetero |
|  |  | *ESK1*target2 | hetero |
|  | 150-3 | *ESK1*target1 | --AGACTGTTCTTTACCCAA |
|  |  | *ESK2*target1 | AGA----GTTCTTTACCCAA |
|  |  | *ESK3*target1 | --AGACTGTTCTTTACCCAA |
|  |  | *ESK1*target2 | ATGCCACTGTGGAA---TAC |
|  | 150-4 | *ESK1*target1 | hetero |
|  |  | *ESK2*target1 | AGA----GTTCTTTACCCAA |
|  |  | *ESK3*target1 | hetero |
|  |  | *ESK1*target2 | hetero |
|  | 150-5 | *ESK1*target1 | hetero |
|  |  | *ESK2*target1 | AGAGA----TCTTTACCCAA |
|  |  | *ESK3*target1 | hetero |
|  |  | *ESK1*target2 | ATGCCA--------------」-------------- |
|  | 150-6 | *ESK1*target1 | hetero |
|  |  | *ESK2*target1 | AGA----GTTCTTTACCCAA |
|  |  | *ESK3*target1 | AGA----GTTCTTTACCCAA |
|  |  | *ESK1*target2 | ATGCCA----------------」------------ |
| *esksgad3* | 150-7 | *ESK1*target1 | --AGACTGTTCTTTACCCAA |
|  |  | *ESK2*target1 | AGA----GTTCTTTACCCAA |
|  |  | *ESK3*target1 | --AGACTGTTCTTTACCCAA |
|  |  | *ESK1*target2 | ATGCCACTGTGGAA---TAC |
|  | 150-8 | *ESK1*target1 | hetero |
|  |  | *ESK2*target1 | AGA----GTTCTTTACCCAA |
|  |  | *ESK3*target1 | hetero |
|  |  | *ESK1*target2 | ATGCCA--------------」-------------- |
|  | 150-9 | *ESK1*target1 | hetero |
|  |  | *ESK2*target1 | AGAGA----TCTTTACCCAA |
|  |  | *ESK3*target1 | hetero |
|  |  | *ESK1*target2 | hetero |
|  | 150-10 | *ESK1*target1 | hetero |
|  |  | *ESK2*target1 | AGAGA----TCTTTACCCAA |
|  |  | *ESK3*target1 | hetero |

**Supplementary Table. 3 Sequences of EG Line 2 sibling.**

| line | Sample name | Target gene | Sequence |
| --- | --- | --- | --- |
| *esksgad3* (Sibling of Line 2) | 127-9-1 | *ESK2*target1 | hetero |
|  | 127-9-2 |  | AGA------TCTTTACCCAA |
|  | 127-9-3 |  | AGA------TCTTTACCCAA |
|  | 127-9-4 |  | AGAAGACTCTTCTTTACCCAA |
|  | 127-9-5 |  | hetero |
|  | 127-9-6 |  | hetero |
|  | 127-9-7 |  | hetero |
|  | 127-9-8 |  | hetero |
|  | 127-9-9 |  | hetero |
|  | 127-9-10 |  | AGA------TCTTTACCCAA |
|  | 127-9-14 |  | AGA------TCTTTACCCAA |
|  | 127-9-18 |  | hetero |
|  | 127-9-26 |  | AGA------TCTTTACCCAA |
|  | 127-9-29 |  | AGA------TCTTTACCCAA |
|  | 127-9-30 |  | hetero |
|  | 127-9-31 |  | hetero |

**Supplementary Table 4. Results of off-target**

| line | Sample name | Off-target candidate region | Sequence | Mutation |
| --- | --- | --- | --- | --- |
| *esksgad3* | 127-5  (Line1) | GAD3-off | CCCGAATCCAAACAAATGGGGG | N |
|  |  | ESKtarget1-off1 | TTGGAGAAAGAACAGTTCT | N |
|  |  | ESKtarget1-off2 | AAAGACTGTTCTTTGAGCCAA | N |
|  |  | ESKtarget1-off3 | TTGGGGAAAGAACAGTACT | N |
|  |  | ESKtarget1-off4 | TTGGGGAAAGAACAGTACT | N |
|  |  | ESKtarget1-off5 | AAGACTGTTCTTTGCCCCA | N |
|  |  | ESKtarget2-off1 | ATGCCATTGTGGAATTTAA | N |
|  |  | ESKtarget2-off2 | ATGCCATTGTGGAATTTAA | N |
|  | 127-9  (Line 2) | GAD3-off | CCCGAATCCAAACAAATGGGGG | N |
|  |  | ESKtarget1-off1 | TTGGAGAAAGAACAGTTCT | N |
|  |  | ESKtarget1-off2 | AAAGACTGTTCTTTGAGCCAA | N |
|  |  | ESKtarget1-off3 | TTGGGGAAAGAACAGTACT | N |
|  |  | ESKtarget1-off4 | TTGGGGAAAGAACAGTACT | N |
|  |  | ESKtarget1-off5 | AAGACTGTTCTTTGCCCCA | N |
|  |  | ESKtarget2-off1 | ATGCCATTGTGGAATTTAA | N |
|  |  | ESKtarget2-off2 | ATGCCATTGTGGAATTTAA | N |

**Supplementary Table 5. List of primers and templates to amplify DNA by PCR.**

| No. | Template | Primer_forward | Sequence(5`→3`) | Primer_reverse | Sequence(5`→3`) |
| --- | --- | --- | --- | --- | --- |
| 1 | pBYR2HS-U6GAD | pPcUf-AtU6pro-F | ccaagctccaattagggccc cgtacgaccctcctgcagttcc | space-U6end-R | tgctagactagtcggttcg cgcagatttgcatccaaaac |
| 2 | PRI201-AN | Nost-F | atcccgggagtgatcgttc aaacatttggcaataaag | PcUbi-Nost-R | cgttgctagggcgcgccga tctagtaacatagatgacacc |
| 3 | SlESK-target2 | space-U6-26-F | ccgactagtctagcaagc ttcctttttttcttcttcttcgttc | SlHWS-target6R | ctcaagcagatcatcccat tcaatcactacttcgactctagc |
| 4 | SlESK-target2 | SlHWS-target6F | aatgggatgatctgcttgagg ttttagagctagaaatagcaag | Nost-U6-endR | gatcactcccgggatttcg cgcagatttgcatccaaaac |
| 5 | pPcUf-SlESKGAD3 | XmaⅠ-U6(26)-F | tgcgcgaaatcccgg ctttttttcttcttcttcgttc | U6-ESKtarget1-R | agagactgttctttacccaac aatcactacttcgactctagc |
| 6 | pPcUf-SlESKGAD3 | ESKtarget1-gRNA-F | ttgggtaaagaacagtctct gttttagagctagaaatagcaag | U6end-XmaI-R | aacgatcactcccgggttcg cgcagatttgcatccaaaac |

**Supplementary Table 6. List of primers to amplify DNA region from tomato genome.**

| No. | Primer_Forward | Sequnece(5`→3`) | Primer_reverse | Sequence(5`→3`) |
| --- | --- | --- | --- | --- |
| 1 | SlGAD3-multiF | cccgaaccctagcagatcgtcttg | SlGAD3-multiR | tcttccaaaactcagcaattgccc |
| 2 | ESK1-target2-multinaF | cagtacacgtaccccttgataga | ESK1-target2-multinaR | caggcatgatgatcctgttcaat |
| 3 | ESK2-target2-multinaF | ctcatataaaataagaccgaggg | ESK2-target2-multinaR | caggcatgatgattctgttcaag |
| 4 | ESK3-target2-multinaF | attcttttcaaatctacttatgg | ESK3-target2-multinaR | tgattctggcatgatgagtctgg |
| 5 | ESK1-target1-multinaF | gaggactccatgtatcagaactg | ESK1-target1-multinaR | caagcagcaactttgctttaaac |
| 6 | ESK2-target1-multinaF | gttacttgcttgaggaatggaag | ESK2-target1-multinaR | cgtaatagctgacaaatacctcc |
| 7 | ESK3-target1-multinaF | gtttttgataatgttactcatcc | ESK3-target1-multinaR | agtaaaacagatagatcgaaacg |
| 8 | M13-47 | cgccagggttttcccagtcacgac | RV-P | ggaaacagctatgaccatgattac |
| 9 | PrimerCheck11-F | gttttcccagtcacgacgtt | Nos-seqR | accggcaacaggattcaatc |
| 10 | PcUbiCheck2-F | tcagatcccctcttccttca | fcoCAS9Check2-R | gtgtccttggagagctggag |
| 11 | fcoCAS9Check3-F | atcctcctctccgacatcct | fcoCAS9Check3-R | agcttctcgttctggagctg |
| 12 | fcoCAS9Check3.5-F | gcgacatgtacgtcgaccag | fcoCAS9Check3.5-R | gacggagtaggcgacggtag |
| 13 | fcoCAS9Check4-F2 | atcaccatcatggagcgctc | fcoCAS9Check4-R2 | acttgtggccgtttacgtcg |
| 14 | gfbsd2Check5-F | cacatgaagcagcacgactt | CaMV35sCheck5-R | gatttcagcgtgtcctctcc |
| 15 | NPTIICheck6-F | agacaatcggctgctctgat | PpcUfcoCas9Check6-R | gcaacgctctgtcatcgtta |
| 16 | AminoPhoCheck7-F | gaaagctgcctgttccaaag | pBR322Check7-R | agctcactcaaaggcggtaa |
| 17 | pBR322Check8-F | gagtcagtgagcgaggaagc | pVS1Check8-R | gattggatgtaccgcgagat |
| 18 | pVS1Check9-F | ggtatcggttcatggattcg | pVS1Check9-R | gagctgcccattcttgagtc |
| 19 | pVS1Check10-F | acccgcctttctggttaagt | PrimerCheck10-R | ctggcgtaatagcgaagagg |
| 20 | HWS-offcheck-1F | tatgttggcagctggccttg | HWS-offcheck-1R | tgcagtgtagccacgtacag |
| 21 | GAD3-offcheck-1F | accagccgagatcattaggc | GAD3-offcheck-1R | tgccacttcctcaatggtcc |
| 22 | ESKtar1-offcheck-1F2 | gagtttgagaaggatggaggc | ESKtar1-offcheck-1R2 | gccattgccatatcatcagcc |
| 23 | ESKtar1-offcheck-2F | ccacagagtctgaagaaacagg | ESKtar1-offcheck-2R | ctgattccaatgccaatgc |
| 24 | ESKtar1-offcheck-3F | gaaccatgatctagggctcg | ESKtar1-offcheck-3R | tgatggtggaaactcccacc |
| 25 | ESKtar1-offcheck-4F | atggttgccctaacttggag | ESKtar1-offcheck-4R | accttgatgcaccaggatcc |
| 26 | ESKtar1-offcheck-5F | aactctctggcttctctgcc | ESKtar1-offcheck-5R | gcagaacaagtagctcccag |
| 27 | ESK1tar2-offcheck-1F | gtggtactcgaacctcctcc | ESK1tar2-offcheck-1R | tcatcacgtgcaatccaagc |
| 28 | ESK1tar2-offcheck-2F | tggtactcgaacctcctccg | ESK1tar2-offcheck-2R | gtaccaatggaagacccagc |
| 29 | *SlNCED*1-F | caacagcaaggaagaagacg | *SlNCED1*-R | tgcctccaacttcaaactca |

**Supplementary Figure 1.** Comparison of *GAD3* and *ESKs* amino acid among Arabidopsis, wild-type tomato and mutants. And *ESK1* comprises three known domains: Transmembrane helix (TM; green), Trichome birefringence-like (TBL; blue), and DUF231 (Red). Highlight indicates motifs.

***AtGAD3* and *SlGAD3* amino acid comparison**

AtGAD3-WT MVLSKTASKSDDSIHSTFASRYVRNSISRFEIPKNSIPKEAAYQIINDELKFDGNPRLNL 60

SlGAD3-WT MVLSK--TPSDDSVHSTFASRYVRTSLPRFEMLEKSIPKEAAYQMINDELMLDGNPRLNL 58

SlGAD3-EG1 MVLSK--TPSDDSVHSTFASRYVRTSLPRFEMLEKSIPKEAAYQMINDELMLDGNPRLNL 58

SlGAD3-EG2 MVLSK--TPSDDSVHSTFASRYVRTSLPRFEMLEKSIPKEAAYQMINDELMLDGNPRLNL 58

***** : ****:**********.*: ***: ::*********:***** :********

AtGAD3-WT ASFVTTWMEPECDKLMMESINKNNVEMDQYPVTTDLQNRCVNMIARLFNAPLGDGEAAIG 120

SlGAD3-WT ASFVTTWMEPECDKLMMASINKNYVDMDEYPVTTELQNRCVNMIARLFNAPLKEEEIGIG 118

SlGAD3-EG1 ASFVTTWMEPECDKLMMASINKNYVDMDEYPVTTELQNRCVNMIARLFNAPLKEEEIGIG 118

SlGAD3-EG2 ASFVTTWMEPECDKLMMASINKNYVDMDEYPVTTELQNRCVNMIARLFNAPLKEEEIGIG 118

***************** ***** *:**:*****:***************** : * .**

AtGAD3-WT VGTVGSSEAVMLAGLAFKRQWQNKRKALGLPYDRPNIVTGANIQVCLEKFARYFEVELKE 180

SlGAD3-WT VGTVGSSEAIMLAGLAFKRNWQNKRKAEGKPYDKPNIVTGANVQVCWEKFANYFEVELKQ 178

SlGAD3-EG1 VGTVGSSEAIMLAGLAFKRNWQNKRKAEGKPYDKPNIVTGANVQVCWEKFANYFEVELKQ 178

SlGAD3-EG2 VGTVGSSEAIMLAGLAFKRNWQNKRKAEGKPYDKPNIVTGANVQVCWEKFANYFEVELKQ 178

*********:*********:******* * ***:********:*** ****.*******:

AtGAD3-WT VKLREGYYVMDPDKAVEMVDENTICVVAILGSTLTGEFEDVKLLNDLLVEKNKKTGWDTP 240

SlGAD3-WT VKLSEGYYVMDPIKAVEMVDDNTICVAAILGSTLNGEFEDVKLLNDLLIEKNKQTGWDTP 238

SlGAD3-EG1 VKLSEGYYVMDPIKAVEMVDDNTICVAAILGSTLNGEFEDVKLLNDLLIEKNKQTGWDTP 238

SlGAD3-EG2 VKLSEGYYVMDPIKAVEMVDDNTICVAAILGSTLNGEFEDVKLLNDLLIEKNKQTGWDTP 238

*** ******** *******:*****.*******.*************:****:******

AtGAD3-WT IHVDAASGGFIAPFLYPDLEWDFRLPLVKSINVSGHKYGLVYAGIGWVVWRTKTDLPDEL 300

SlGAD3-WT IHVDAASGGFIAPFIYPELEWDFRLPLVKSINVSGHKYGLVYAGIGWVIWRTKQDLPQQL 298

SlGAD3-EG1 IHVDAASGGFIAPFIYPELEWDFRLPLVKSINVSGHKYGLVYAGIGWVIWRTKQDLPQQL 298

SlGAD3-EG2 IHVDAASGGFIAPFIYPELEWDFRLPLVKSINVSGHKYGLVYAGIGWVIWRTKQDLPQQL 298

**************:**:******************************:**** ***::*

AtGAD3-WT IFHINYLGADQPTFTLNFSKGSSQVIAQYYQLIRLGFEGYRNVMDNCRENMMVLRQGLEK 360

SlGAD3-WT IFHINYLGADQPTFTLNFSKGSSQVIAQYYQLIRLGYEGYRNVMENCRENAIVLRKGLEK 358

SlGAD3-EG1 IFHINYLGADQPTFTLNFSKGSSQVIAQYYQLIRLGYEGYRNVMENCRENAIVLRKGLEK 358

SlGAD3-EG2 IFHINYLGADQPTFTLNFSKGSSQVIAQYYQLIRLGYEGYRNVMENCRENAIVLRKGLEK 358

************************************:*******:***** :***:****

AtGAD3-WT TGRFNIVSKENGVPLVAFSLKDSSRHNEFEVAEMLRRFGWIVPAYTMPADAQHVTVLRVV 420

SlGAD3-WT TGRFNIISKDEGIPLVAFSLKDNSLHNEFEVSETLRRFGWIVPAYTMPADLQHVTVLRVV 418

SlGAD3-EG1 TGRFNIISKDEGIPLVAFSLKDNSLHNEFEVSETLRRFGWIVPAYTMPADLQHVTVLRVV 418

SlGAD3-EG2 TGRFNIISKDEGIPLVAFSLKDNSLHNEFEVSETLRRFGWIVPAYTMPADLQHVTVLRVV 418

******:**::*:*********.* ******:* **************** *********

AtGAD3-WT IREDFSRTLAERLVADFEKVLHELDTLPARVHAKMASGKVNGVKKTPEETQREVTAY**W**KK 480

SlGAD3-WT IREDFSRTLADRLVSDIVKVLHELPNAKKVEDN----LMINNEKKTEIEVQRAIAEF**W**KK 474

SlGAD3-EG1 IREDFSRTLADRLVSDIVKVLHELPNAKWRII-------------------------**-**-- 450

SlGAD3-EG2 IREDFSRTLADRLVSDIVKVLHELPNAKKVGG-------------------------**-**-- 450

**********:***:*: ****** . CaMBD binding motif

AtGAD3-WT FVDTKTDKNGVPLVASITNQ 500

SlGAD3-WT YVLARKASIC---------- 484

SlGAD3-EG1 -------------------- 450

SlGAD3-EG2 -------------------- 450

***AtESK1* and *SlESK1* amino acid comparison**

AtESK1-WT --MQPWRRKFPLFETG----VTMKQRKNSNLSIFVVVFSVFLFGIFMYNEDVKSIAEFPF 54

SlESK1-WT MYSSLRSRKTPLFFSSNLLKMKHNARKNNHFSIFVVVFSIFLFGCFMYNEDVKTIAEFPF 60

SlESK1-EG1/2 MYSSLRSRKTPLFFSSNLLKMKHNARKNNHFSIFVVVFSIFLFGCFMYNEDVKTIAEFPF 60

. ** *** :. :. : ***.::********:**** ********:******

AtESK1-WT STSKPHDVHDEATPITEITTLPVQESIKNSDPIQESIKNADSVQDSVKDVAEPVQEEVSK 114

SlESK1-WT SMTRNQDIYTPPLN--QQ------N----------------GVQEIETKVVMNSRTEQET 96

SlESK1-EG1/2 SMTRNQDIYTPPLN--QQ------N----------------GVQEIETKVVMNSRTEQET 96

* :: :*:: : : .**: ..*. : * ..

AtESK1-WT TEEVKKIELFA-ATEDEEDVELPPEECDLFTGEWVFDNETHPLYKEDQCEFLTAQVTCMR 173

SlESK1-WT EQEKEKIEIPAEEEEEEESIELPPEDCDLFTGQWVYDNVTHPVYKEPECEFLTAQVTCMR 156

SlESK1-EG1/2 EQEKEKIEIPAEEEEEEESIELPPEDCDLFTGQWVYDNVTHPVYKEPECEFLTAQVTCMR 156

:* :***: * *:**.:*****:******:**:** ***:*** :************

AtESK1-WT NGRRDSLYQNWRWQPRDCSLPKFKAKLLLEKLRNKRMMFVGDSLNRNQWESMVCLVQSVV 233

SlESK1-WT NGREDSMYQNWRWQPRDCSLPKFKAKLLLEKLRNKRLMFVGDSLNRNQWESMICLVQSAV 216

SlESK1-EG1/2 NGREDSMYQNWRWQPRFFTQV--------------------------------------- 177

***.**:********* : GDS motif

AtESK1-WT PPGRKSLNKTGSLSVFRVEDYNATVEFYWAPFLVESNSDDPNMHSILNRIIMPESIEKHG 293

SlESK1-WT PSGRKSLNKTGSLSVFRIEDYNATVEFYWAPFLVESNSDDPNMHSILNRIIMPESIEKHG 276

SlESK1-EG1/2 ------------------------------------------------------------ 177

AtESK1-WT VNWKGVDFLVFNTYIWWMNTFAMKVLRGSFDKGDTEYEEIERPVAYRRVMRTWGDWVERN 353

SlESK1-WT KNWKNVDYLVFNTYIWWMNTFAMKVLRGSFDEGATEYDEIERPVAYRRVLTTWSQWVENN 336

SlESK1-EG1/2 ------------------------------------------------------------ 177

AtESK1-WT IDPLRTTVFFASMSPLHIKSLDWENPDGIKCALETTPILNMSMPFSVGTDYRLFSVAENV 413

SlESK1-WT VDVNSTKVFFMSMSPLHIKSLDWNNPDGIKCAKETTPVLNTSMPLSVGTDKRLFVVAANV 396

SlESK1-EG1/2 ------------------------------------------------------------ 177

AtESK1-WT THSLNVPVYFLNITKLSEYRKDAHTSVHTIRQGKMLTPEQQADPNTYADCIHWCLPGLPD 473

SlESK1-WT TQSINVPVYFLNITSLSEYRKDAHTSVHTIRQGKMLTAEQKADPTTYADCIHWCLPGLPD 456

SlESK1-EG1/2 ------------------------------------------------------------ 177

DXXH motif

AtESK1-WT TWNEFLYTRIISRS 487

SlESK1-WT TWNEFLYSRILSHS 470

SlESK1-EG1/2 -------------- 177

***AtESK1* and *SlESK2* amino acid comparison**

AtESK1-WT MQPWRRKFPLFETGVTMKQRKNSNLSIFVVVFSVFLFGIFMYNEDVKSIAEFPFSTSKPH 60

SlESK2-WT --------------MKHGGQKNNNLSIVVVVFSIFLFSCFIYNEDFKSIAEFPFSRPKIQ 46

SlESK2-EG1/2 --------------MKHGGQKNNNLSIVVVVFSIFLFSCFIYNEDFKSIAEFPFSRPKIQ 46

SlESK2-EG (127-4) --------------MKHGGQKNNNLSIVVVVFSIFLFSCFIYNEDFKSIAEFPFSRPKIQ 46

:. :**.****.*****:***. *:****.********* * :

AtESK1-WT DVHDEATPITEITTLPVQESIKNSDPIQESIKNADSVQDSVKDVAEPVQEEVSKTEEVKK 120

SlESK2-WT LESDENRVSPSM--------VMNS-----------------------------RTIVETE 69

SlESK2-EG1/2 LESDENRVSPSM--------VMNS-----------------------------RTIVETE 69

SlESK2-EG (127-4) LESDENRVSPSMV--------MNS-----------------------------RTIVETE 69

** .: ** :* .:

AtESK1-WT IELFAATEDEEDVELPPEECDLFTGEWVFDNETHPLYKEDQCEFLTAQVTCMRNGRRDSL 180

SlESK2-WT IEQSIEMAEDENIELPPDDCDLFTGNWVYDNISHPIYKEDQCEFLTSQVTCLRNGRKDSM 129

SlESK2-EG1/2 IEQSIEMAEDENIELPPDDCDLFTGNWVYDNISHPIYKEDQCEFLTSQVTCLRNGRKDSM 129

SlESK2-EG (127-4) IEQSIEMAEDENIELPPDDCDLFTGNWVYDNISHPIYKEDQCEFLTSQVTCLRNGRKDSM 129

** ::*::****::******:**:** :**:**********:****:****:**:

AtESK1-WT YQNWRWQPRDCSLPK-----FKAKLLLEKLRNKRMMFVG-----DSLNR-NQWESMVCLV 229

SlESK2-WT YQNWRWQPRDCSLPNKKNFRFKPRLLLEKLRNKRLMFVG-----DSLNR-NQWESMVCLV 183

SlESK2-EG1/2 YQNWRWQPR--SLPNKKNFRFKPRLLLEKLRNKRLMFVG-----DSLNR-NQWESMVCLV 181

SlESK2-EG (127-4) YQNWRWQPRRLFFTQ-KEFQV-TKIAS-EIEEQETHVCRRLIEQESMGIHGLFGSICCSF 186

********* : : . :: ::.::. . :*:. . : *: * .

GDS motif

AtESK1-WT QSVVPPGRKSLNKTGSLSVFRVEDYNATVEFYWAPFLVESNS--DDPNMHSILNRIIMPE 287

SlESK2-WT QSVVPSAKKSLNKIGSLSVFTIQDYNATVEFYWAPFLVESNS--DDPNMHSILNRIIMPE 241

SlESK2-EG1/2 QSVVPSAKKSLNKIGSLSVFTIQDYNATVEFYWAPFLVESNS--DDPNMHSILNRIIMPE 239

SlESK2-EG (127-4) CQKKPK----------------------QNWFFICFHNSGLQCHGGILLGSISSRVKLG- 223

. * :::: * .. . .. : ** .*: :

AtESK1-WT SIEKHGVNWKGVDFLVFNTYIWWMNTFAMKVLRGSFDKGDTEYEEIERPVAYRRVMRTWG 347

SlESK2-WT SIDKHGNNWKNVDYLIFNTYIWWMNTFSMKILRGSFDEGATEYDETDRPTAYKRVLTTWS 301

SlESK2-EG1/2 SIDKHGNNWKNVDYLIFNTYIWWMNTFSMKILRGSFDEGATEYDETDRPTAYKRVLTTWS 299

SlESK2-EG (127-4) ------------------------------------------------------------ 223

AtESK1-WT DWVERNIDPLRTTVFFASMSPLHIKSLDWENPDGIKCALETTPILNMSMPFSVGTDYRLF 407

SlESK2-WT QWVDKNIDPNRTQVFFMSMSPLHIKSFDWENPDGIKCAKETSPILNTSMPLNVGTDKRLF 361

SlESK2-EG1/2 QWVDKNIDPNRTQVFFMSMSPLHIKSFDWENPDGIKCAKETSPILNTSMPLNVGTDKRLF 359

SlESK2-EG (127-4) ------------------------------------------------------------ 223

AtESK1-WT SVAENVTHSLNVPVYFLNITKLSEYRKDAHTSVHTIRQGKMLTPEQQADPNTYADCIHWC 467

SlESK2-WT VIAANVIRSIKVPVYFLNITTLSEYRKDAHTSVHTIRQGKMLTPEQQDDPATYADCIHWC 421

SlESK2-EG1/2 VIAANVIRSIKVPVYFLNITTLSEYRKDAHTSVHTIRQGKMLTPEQQDDPATYADCIHWC 419

SlESK2-EG (127-4) ------------------------------------------------------------ 223

DXXH motif

AtESK1-WT LPGLPDTWNEFLYTRIISRS 487

SlESK2-WT LPGLPDTWNEFLYSRIVSHS 441

SlESK2-EG1/2 LPGLPDTWNEFLYSRIVSHS 439

SlESK2-EG (127-4) -------------------- 223

***AtESK1* and *SlESK3* amino acid comparison**

AtESK1-WT MQPWRRKFP----LFET--GVTMKQR-KNSNLSIFVVVFSVFLFGIFMYNEDVKSIAEFP 53

SlESK3-WT MHSFQLKISKIATFFSPPKLFNMKQNCRNSHKSCIILFSIFFFFVFFMYNEDIKSIAQLQ 60

SlESK3-EG1/2 MHSFQLKISKIATFFSPPKLFNMKQNCRNSHKSCIILFSIFFFFVFFMYNEDIKSIAQLQ 60

*: :: *: :*. ..***. :**: * :::. .*:* :******:****::

AtESK1-WT FSTSKPHDVHDEATPITEITTLPVQESIKNSDPIQESIKNADSVQDSVKDVAEPVQEEVS 113

SlESK3-WT FISNPKI-LENSS-------------------------NKSNGVQE----ISKI----LG 86

SlESK3-EG1/2 FISNPKI-LENSS-------------------------NKSNGVQE----ISKI----LG 86

* :. :.:.: ::::.**: ::: :.

AtESK1-WT KTEEVKKIELFAATEDEEDVELPPEECDLFTGEWVFDNETHPLYKEDQCEFLTAQVTCMR 173

SlESK3-WT KQSKNTSVLTNSRSENDDEIELPPESCDLFNGQWVFDNVTHPIYKEKECKFLTEQVTCLK 146

SlESK3-EG1/2 KQSKNTSVLTNSRSENDDEIELPPESCDLFNGQWVFDNVTHPIYKEKECKFLTEQVTCLK 146

* .: ..: : :*:::::*****.****.*:***** ***:***.:*:*** ****::

AtESK1-WT NGRRDSLYQNWRWQPRDCSLPKFKAKLLLEKLRNKRMMFVGDSLNRNQWESMVCLVQSVV 233

SlESK3-WT NGRKDSLFQNWRWQPRDCSLPKFRARLLLEKLRNKRLMFVGDSLNRNQWESMICLLQSGV 206

SlESK3-EG1/2 NGRKDSLFQNWRWQPRFFTQV--------------------------------------- 167

***:***:******** : GDS motif

AtESK1-WT PPGRKSLNKTGSLSVFRVEDYNATVEFYWAPFLVESNSDDPNMHSILNRIIMPESIEKHG 293

SlESK3-WT PFSWNKYKKIGHLSVFIIEKYNATVEFYWAPYLVESNSDDPAVHNILTRLIMPESIEKHG 266

SlESK3-EG1/2 ------------------------------------------------------------ 167

AtESK1-WT VNWKGVDFLVFNTYIWWMNTFAMKVLRGSFDKGDTEYEEIERPVAYRRVMRTWGDWVERN 353

SlESK3-WT NSWKNVDYLIFNTYVWWMNGGTMKVLRGSFDKGSKEYDEIDRTIAYTRVLTTWSQWVQNN 326

SlESK3-EG1/2 ------------------------------------------------------------ 167

AtESK1-WT IDPLRTTVFFASMSPLHIKSLDWENPDGIKCALETTPILNMSMPFSVGTDYRLFSVAENV 413

SlESK3-WT VDPKRTRVFFMSMSPMHIKSMDWNNPNGIKCAKETTPVLNMSMPLNVGTDKRLFDIASNV 386

SlESK3-EG1/2 ------------------------------------------------------------ 167

AtESK1-WT THSLNVPVYFLNITKLSEYRKDAHTSVHTIRQGKMLTPEQQADPNTYADCIHWCLPGLPD 473

SlESK3-WT TKSIKLPVYFMNITRISEYRKDAHTSLYTIRQGKILTPEQQADPATYADCIHWCLPGLPD 446

SlESK3-EG1/2 ------------------------------------------------------------ 167

DXXH motif

AtESK1-WT TWNEFLYTRIISRS 487

SlESK3-WT TWNELLYTRIMSSS 460

SlESK3-EG1/2 -------------- 167

**A**


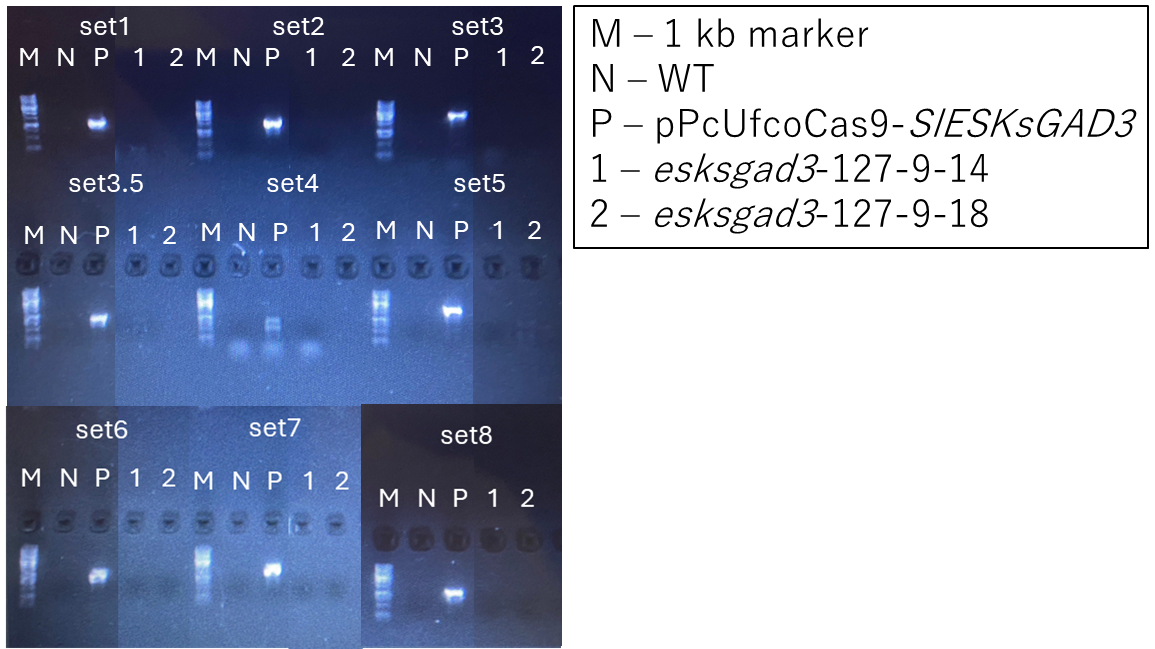


**B**

| Line | Sample name | Result(N,P) |
| --- | --- | --- |
| *esksgad3* | 127-5-3 | P |
|  | 127-5-6 | N |
|  | 127-5-13 | P |
|  | 126-5-14 | P |
|  | 127-5-15 | P |
|  | 127-9-2 | P |
|  | 127-9-4 | P |
|  | 127-9-10 | P |
|  | 127-9-14 | N |
|  | 127-9-18 | N |
|  | 127-9-26 | N |
|  | 127-9-29 | N |
|  | 127-9-30 | P |

**Supplementary Figure 2. (Result of foreign gene check)**

(A) Results of PCR for foreign gene check. M, maker 1kbp; N, negative control (Mili-Q); P, positive control (Vector for targeting *SlESK 1-3* and *SlGAD3*, the bands could be detected.); 1 and 2, samples (the bands couldn’t be detected). (B) Results of foreign gene check. The results were confirmed in the same way as (A). P, positive result of foreign gene; N, negative result of foreign gene. **
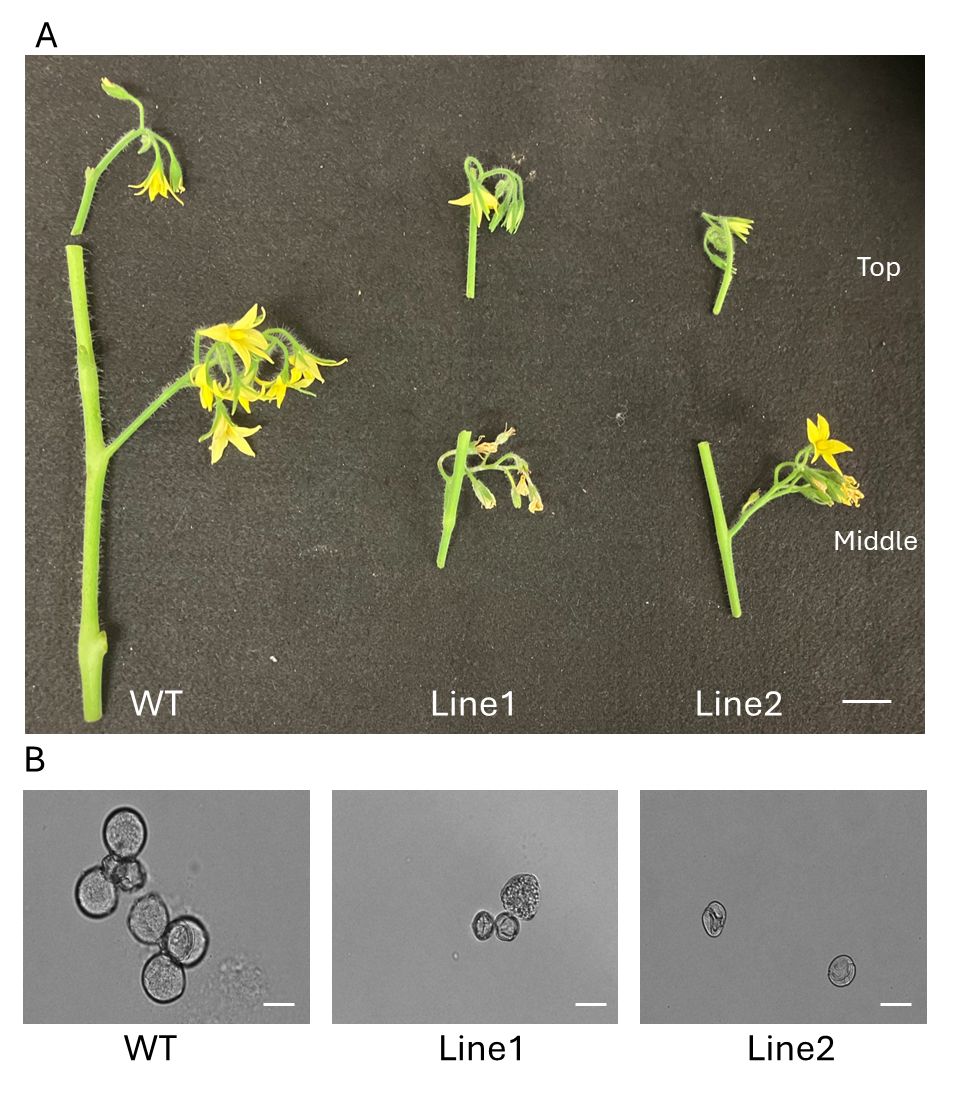
Supplementary Figure 3.** (A) An image of flower in WT and EG lines. Top indicates the flower which is top parts of plants and 10 days old flowers. Middle indicates the flower which is middle parts of plants and 20 days old flowers. (left: WT, middle: EG Line1, right: EG Line 2) (B) Illustrations of pollen in WT and EG lines. The pollens of WT are circled shape and identical size, while the pollens of EG lines are inconsistent shape and size. (Scale bar: 20μm)


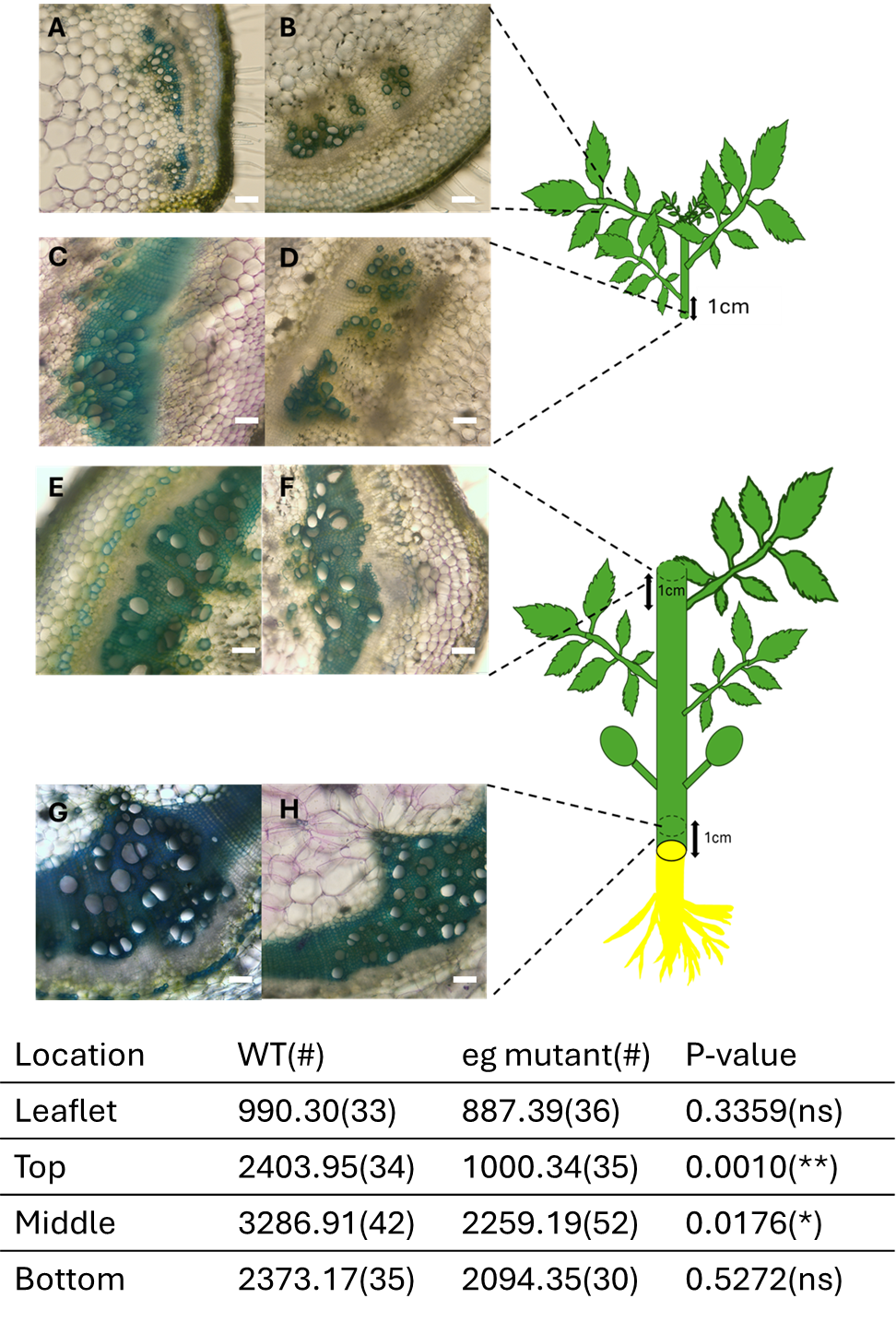

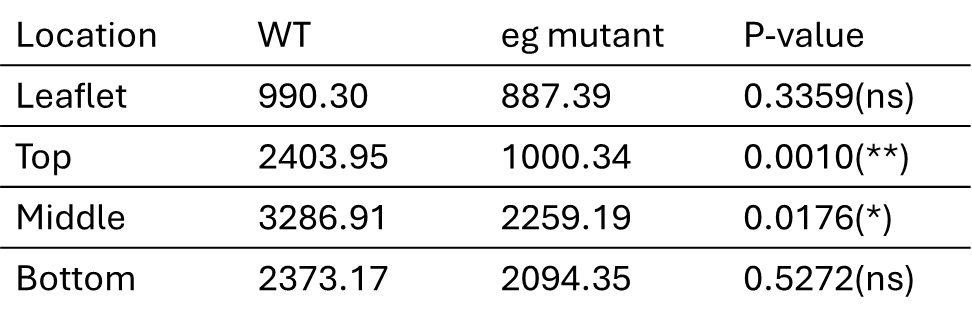


**I**

**Supplementary Figure 4.** (A-H) Xylem of stem (left: WT, right: EG Line 2) (Scale bar: 100μm) (A-B) Xylem of leaflet part. (C-D) Xylem of upper part. (E-F) Xylem of middle part. (G-H) Xylem of above part. (I) Average width of vessel (μm^2^). Statistical significance: ns, no significant difference; *, p < 0.05; *, p < 0.5; **).


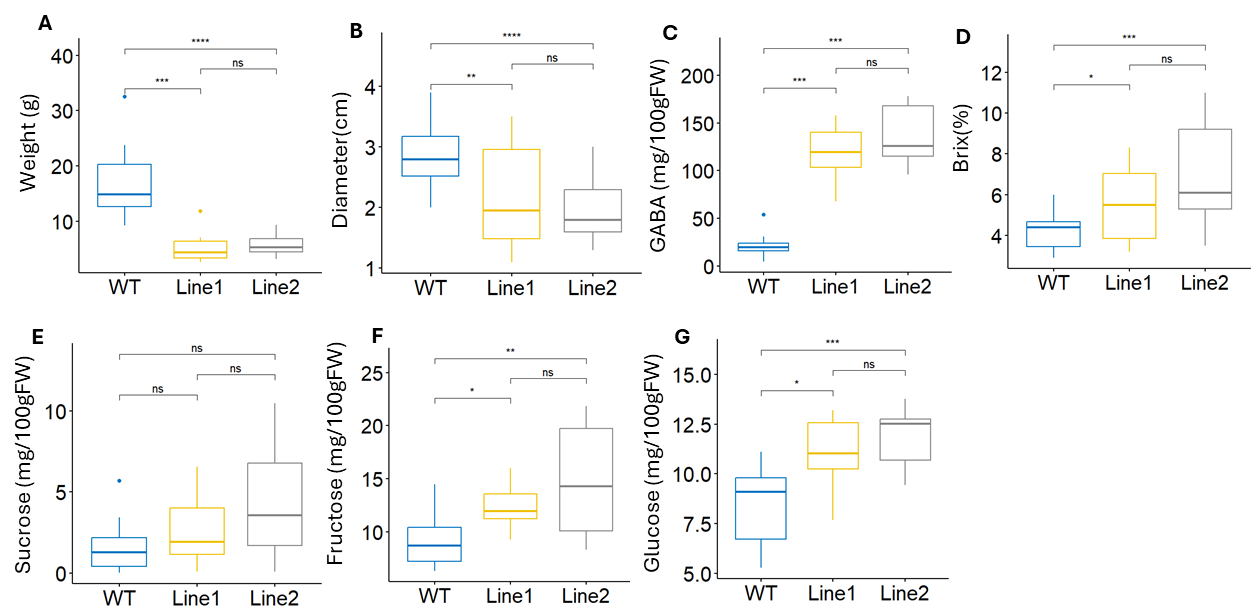


**Supplementary Figure 5. Parameter of fruits from WT and EG Lines (EG Line 1 and EG Line 2) during Summer season by using fruits’ numbers (WT, n = 11; Line 1, n = 8; Line 2, n = 12).**

(A)Fruit weight. (B) Fruit diameter. (C) GABA concentration in fruits. (D) Brix value of fruits. (E) Sucrose concentration in fruits. (F) Fructose concentration in fruits. (G) Glucose concentration in fruits. Statistical significance: ns, no significant difference; *, p < 0.05; *, p < 0.5; **, p < 0.01; ***, p < 0.001; ****, p < 0.0001.


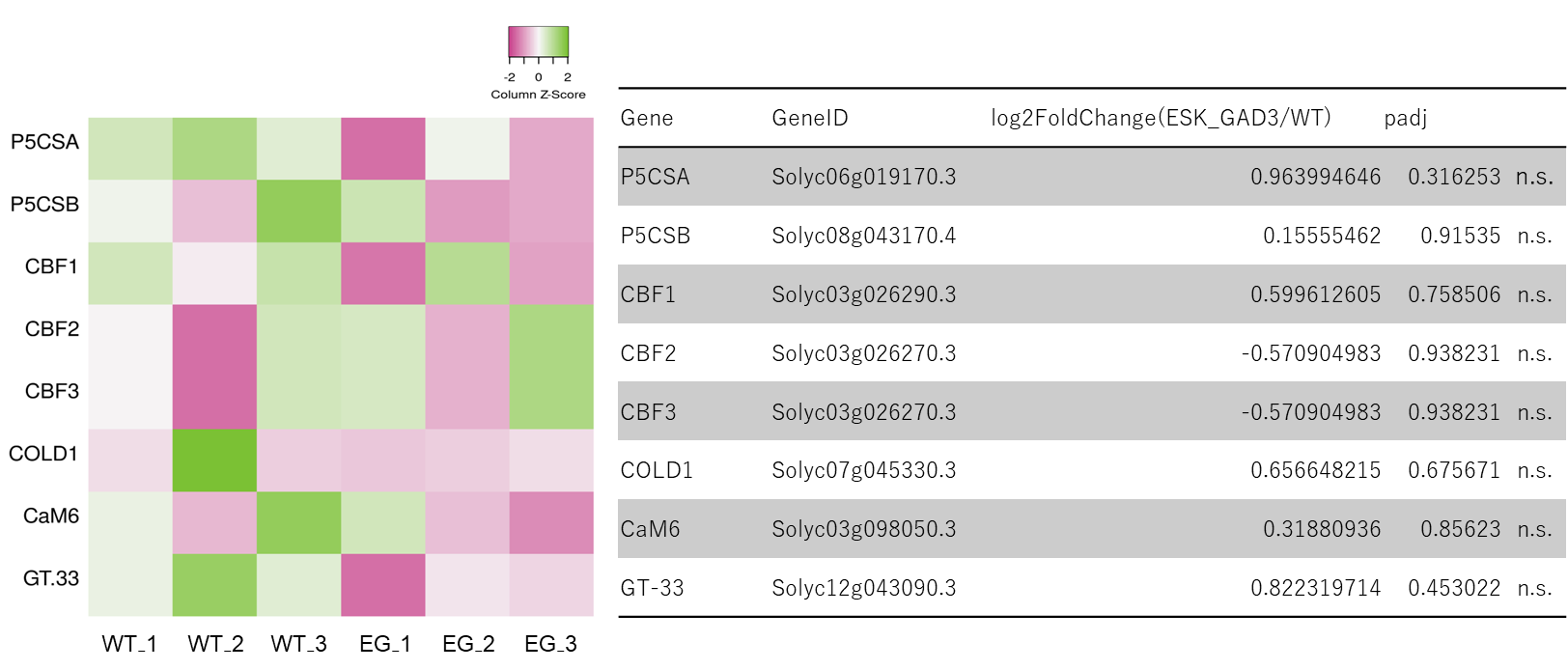

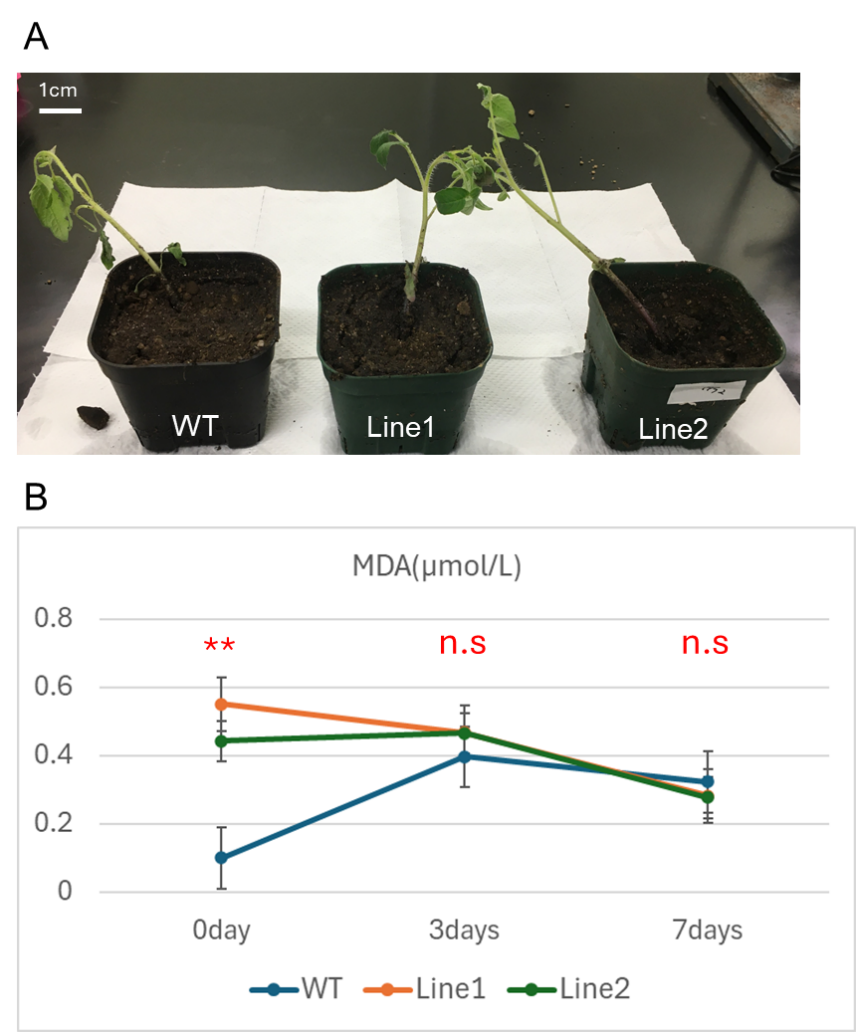
**Supplementary Figure 6. EG Line 1 and Line 2 didn’t exhibit cold stress tolerance (n = 3, each).**

**C**

(A) Illustrates are of representative plants of WT and EG Line 1, EG Line 2 7-days after cold stress treatment. Scale bar represents 1 cm in length. (B) Concentration of MDA before stress (0 day) and after 3-days and 7-days the cold stress treatment. Statistical significance: ns, no significant difference; **, p < 0.01. (C) Heatmap for representative cold-related gene expression by RNA-seq for differentially expressed genes (DEGs) between WT and EG Line 2 in room temperature condition. Green is higher expression and pink is lower expression; DEGs defined as genes with a log base two fold change value less than -1 or greater than 1 and FDR adjusted p-value less than 0.05; n.s., no significant difference.


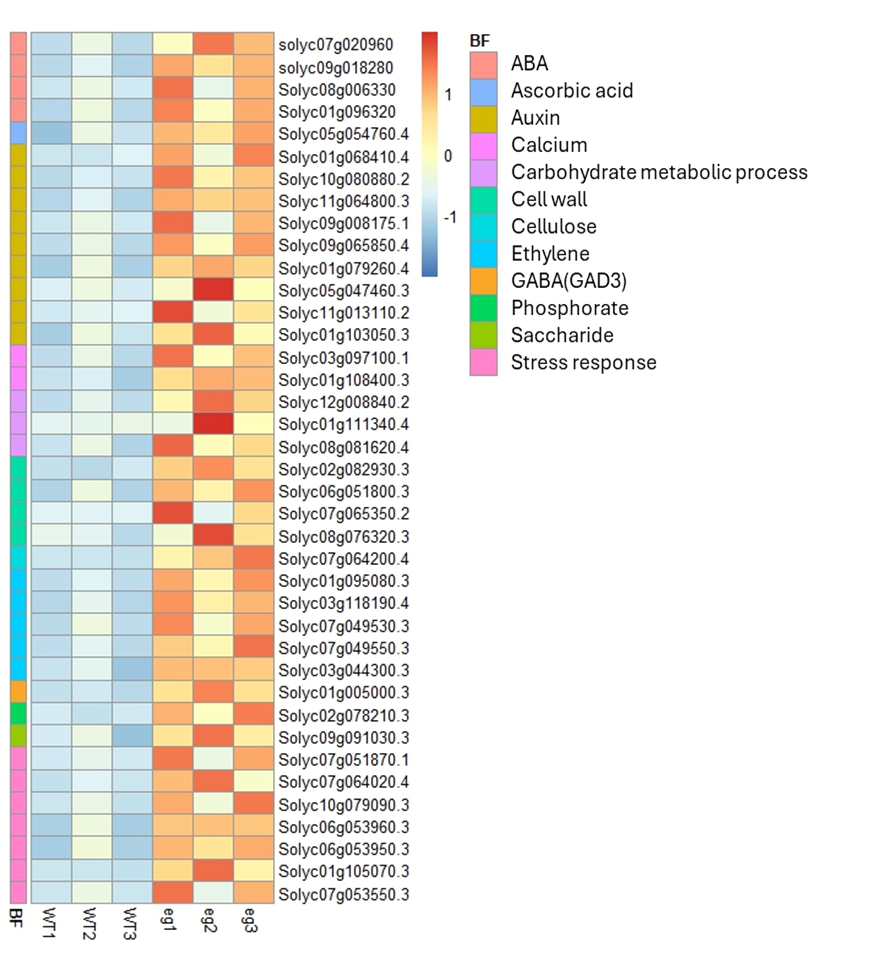


**Supplementary Figure 7.** Heatmap for representative up-regulated gene expression by RNA-seq for differentially expressed genes (DEGs) between WT and EG Line 2. Red is higher expression and blue is lower expression; DEGs defined as genes with a log base two fold change value less than -1 or greater than 1 and FDR adjusted p-value less than 0.05. BF represents biological function.


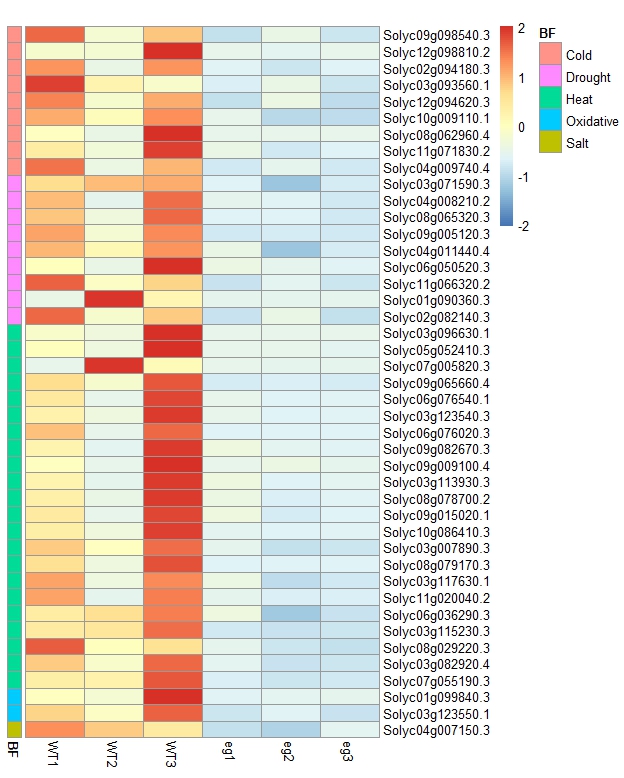

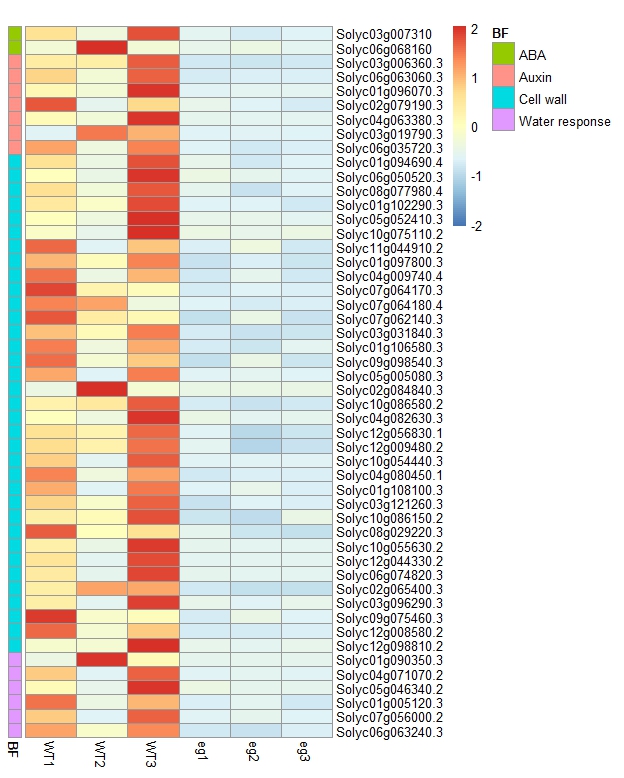

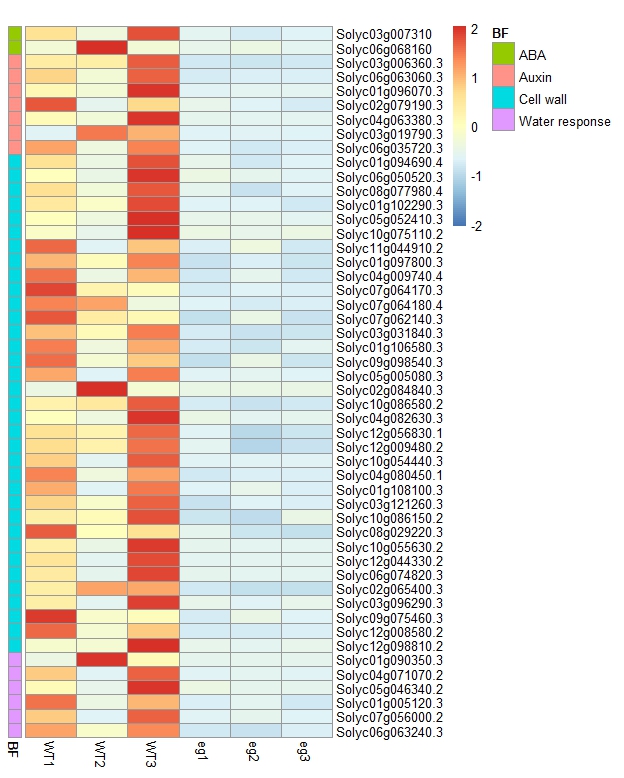

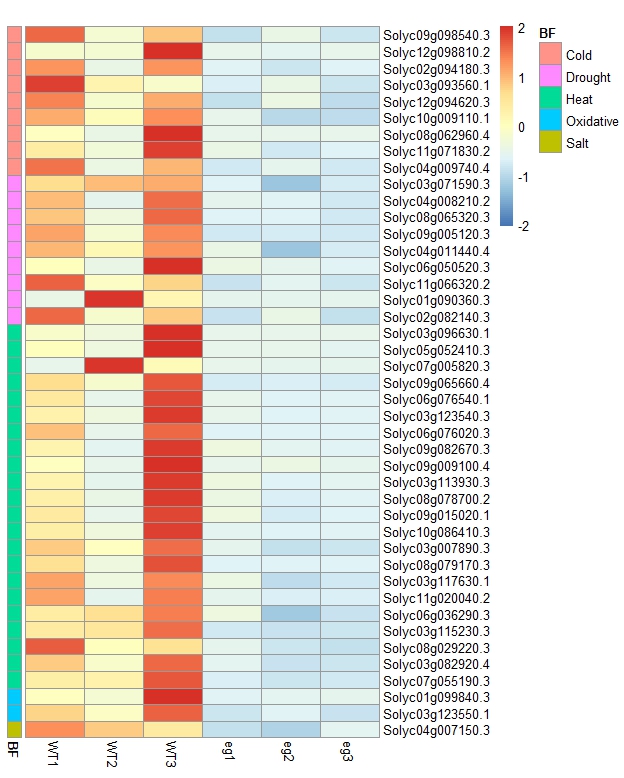


**Supplementary Figure 8.** Heatmap for representative down-regulated gene expression by RNA-seq for differentially expressed genes (DEGs) between WT and EG Line 2. Red is higher expression and blue is lower expression; DEGs defined as genes with a log base two fold change value less than -1 or greater than 1 and FDR adjusted p-value less than 0.05. BF represents biological function.
